# Is There a Future Without Gluten Restrictions for Celiac Patients? Update on Current Treatments

**DOI:** 10.3390/nu17182960

**Published:** 2025-09-15

**Authors:** Marina Girbal-González, Francisco J. Pérez-Cano

**Affiliations:** 1 Physiology Section, Department of Biochemistry and Physiology, Faculty of Pharmacy and Food Science, University of Barcelona (UB), 08028 Barcelona, Spain; marinagirbal@ub.edu; 2 Nutrition and Food Safety Research Institute (INSA-UB), University of Barcelona (UB), 08921 Santa Coloma de Gramenet, Spain

**Keywords:** celiac disease (CeD), gluten, gluten-free diet, gliadin, glutenase, treatments, non-celiac gluten/wheat sensitivity, probiotics, nutraceuticals

## Abstract

Celiac disease (CeD) is a chronic autoimmune enteropathy triggered by dietary gluten in genetically predisposed individuals. Along with other disorders such as non-celiac gluten/wheat sensitivity and gluten allergy, adherence to a strict gluten-free diet (GFD) is required as the only effective treatment for CeD. To this end, and partially due to the burdensome nature and limited efficacy in some patients of a GFD, significant research into alternative therapies has been catalyzed. This review gives a perspective on current and emerging treatment strategies targeting different aspects of CeD pathogenesis. These include gluten-degrading enzymes (e.g., AN-PEP, Latiglutenase, Zamaglutenase), gluten-sequestering agents (e.g., AGY-010, BL-7010), modulators of intestinal permeability (e.g., Larazotide acetate, IMU-856), immune-modulating agents (e.g., ZED1227, AMG 714, EQ102), and strategies for immune tolerization (e.g., TAK-101, KAN-101, Nexvax2). Newer approaches are also targeting probiotics to modulate the gut microbiota (e.g., VSL#3, *Lactobacillus plantarum* HEAL9), nutraceuticals (e.g., polyphenols, vitamins), or food modifications to remove the gluten from naturally gluten-containing foodstuffs (e.g., gluten transamidation, Gluten Friendly™ technology). Despite encouraging results in preclinical and clinical trials, no treatment has yet been conclusively proven to serve as an effective alternative to the GFD. Continued research is essential to validate efficacy, optimize dosing, and ensure safety in broader patient populations. Here, we provide a comprehensive overview of the therapeutic landscape for CeD, analyze the main strengths and limitations of each treatment and highlight promising directions for future management of CeD, altogether evidencing the urgent need to develop effective alternatives for these patients.

## 1. Celiac Disease Pathophysiology and Limitations of a Gluten-Free Diet

Celiac disease (CeD) is a chronic autoimmune enteropathy mediated by exposure to dietary gluten in genetically predisposed individuals [1,2]. Gluten encompasses a group of related storage proteins found mainly in wheat and in other cereals including rye, barley and oat. In wheat, the main proteins are gliadin and glutenin, which belong to the prolamin family and contain high amounts of glutamine and proline residues. These can resist degradation by regular digestive enzymes, which can mediate the immune reaction triggered after gluten intake in the context of CeD [3]. One of the most immunoreactive peptides in CeD is 33-mer, resulting from degradation of α2-gliadin by intestinal enzymes and which contains three T-cell-reactive epitopes [4]. Such partially digested peptides, also named gluten immunogenic peptides (GIPs), are mainly transported through transcellular pathways and reach the lamina propria, where they can be deamidated by the tissue transglutaminase 2 enzyme (tTG2 or TG2). Then, either these deamidated gluten peptides (DGPs), alone or complexed to tTG2, can bind to the Human leukocyte antigen (HLA)-DQ2 or DQ8 molecules in antigen-presenting cells (APCs). These activate gluten-specific CD4+ T cells, which leads to the development of tTG2- and gluten-specific B cells that evolve into anti-tTG2 and anti-DGP antibody-producing plasma cells. Simultaneously, an overall inflammatory milieu is created through secretion of interleukin (IL)-15 by APCs and epithelial cells or IL-21 and interferon (IFN)-ɣ by activated CD4+ T cells, among others. These pro-inflammatory cytokines also promote infiltration of lymphocytes into the gut epithelium (IELs) and cause intestinal atrophy [5].

The worldwide prevalence of CeD ranges from 1.1 to 1.7% [6], and it affects more women than men in a 1.79:1 ratio [7]. The seroprevalence of CeD is higher in Europe and Asia, and it is thought to be able to increase in the coming years since most patients remain undiagnosed and new screening policies are being implemented [8]. Nevertheless, the only currently effective treatment for CeD is strict adherence to a GFD, which can be difficult to maintain and poses a great socioeconomic burden [9]. Hence, finding a treatment for CeD is presently a very active line of research in the biomedical field. However, even with this commitment, many of the investigations performed and technologies developed failed to finally reach patients and help in day-to-day life.

**Figure 1:**
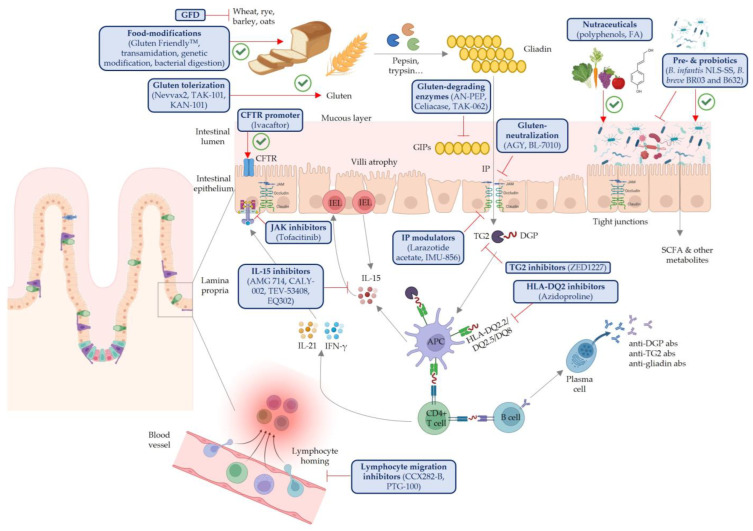
Pathophysiology of CeD and site of action inhibited, modulated or promoted by all the existent therapeutic approaches to prevent, cure or ameliorate symptomatology of this disease. Current treatments can be classified into seven main groups based on their targeted step within the gluten-derived immunological cascade: gluten tolerization, gluten-degrading enzymes, gluten neutralization, IP modulators, immune modulators (CFTR promoters, TG2, IL-15, HLA-DQ2, JAK or lymphocyte migration inhibitors), pre-, pro- and synbiotics, nutraceuticals and modification of gluten-containing foodstuffs. The only currently accepted treatment for CeD is a GFD.

In this perspective, we aim to thoroughly present all the current approaches to prevent or treat CeD. Although this topic is being approached from many different, sometimes complementary, outlooks, the options that are currently under research fall into eight main categories based on their mechanism of action. First, gluten-degrading enzymes, one of the most promising strategies, aim to digest gliadin and GIPs to obtain smaller peptides, which can then potentially go unrecognized by the immune system. Alternatively, gluten-sequestering or -neutralizing compounds can complex to gluten or gliadin and prevent them from crossing into the lamina propria. Many kinds of immune modulators have also been investigated, which intend to stop the gluten-derived immune cascade at different stages. For example, TG2 inhibitors try to prevent deamidation of GIPs and the subsequent interaction with the HLA-DQ2/8 receptors in CD4+ T cells. Other strategies within this category include IL-15 inhibitors, which aim to prevent the establishment of an inflammatory milieu and IEL infiltration, or HLA-DQ2 inhibitors, to prevent interaction between these and deamidated GIPs. Gluten tolerization has also been researched with the objective to prevent an immune response to gluten ingestion altogether. Moreover, since gut dysbiosis is a hallmark of CeD, two alternative strategies, i.e., promotion of intestinal integrity and gut microbiota modulation, have been targeted through intestinal permeability (IP) modulators and pro-, pre-, synbiotics and fecal microbiota transplantation (FMT), respectively. The working hypothesis for these treatments is that an improvement in overall gut health might help decrease the CeD-associated symptoms. Simultaneously, the use of a wide range of naturally derived bioactive compounds (nutraceuticals) has also been researched to prevent or ameliorate CeD symptomatology, since this can exert anti-inflammatory, antioxidant and prebiotic actions. Finally, several strategies have been directed at achieving gluten-free foods from gluten-containing cereals, through modification of gliadin, breeding strategies or enzymatic degradation. A schematic overview of CeD pathophysiology and the mechanistic target of all the reviewed therapeutic agents is shown in Figure 1.

Initially, the inclusion criteria for the current perspective comprised scientific articles primarily published within the last ten years, which were identified through a comprehensive search of the literature and subsequently categorized according to the treatment field (e.g., gluten-degrading enzymes, gluten neutralization strategies, immune-modulating therapies). However, given our aim to provide an integrative overview of approaches that have failed, evolved, or remain under active development, earlier publications predating this timeframe, including seminal studies foundational to current research directions, were also considered.

## 2. Gluten-Degrading Enzymes

**Figure 2:**
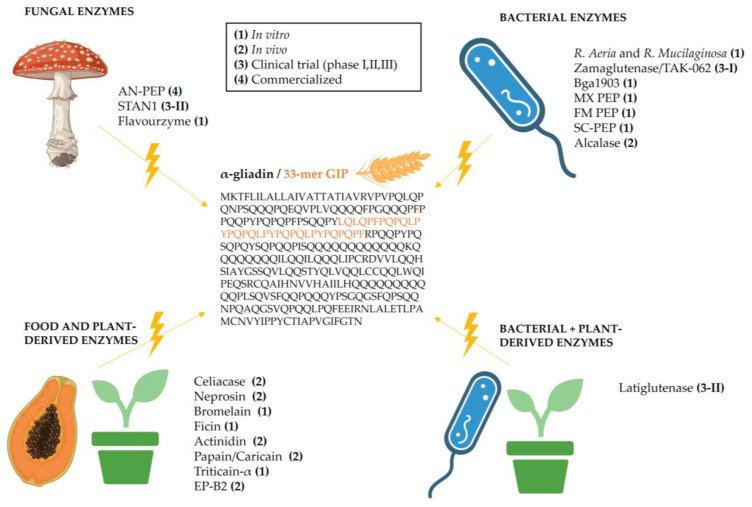
Current research on gluten-degrading enzymes, origin and research status: (**1**) in vitro, (**2**) in vivo, (**3**) clinical trial or (**4**) commercialized.

This approach has been researched for over 20 years, and multiple clinical trials are still going on. For an enzyme to be considered a good therapeutic option for CeD, it must meet several criteria simultaneously: (1) efficiently target and break down the proline- and glutamine-rich peptides, (2) remain active in the acidic environment of the stomach (low pH), (3) resist proteolysis by digestive enzymes, (4) be active at the body’s temperature and (5) be non-toxic and non-immunogenic [10]. Active, completed and terminated trials, as well as in vitro and preclinical studies on the topic, are summarized in Table 1 and Figure 2.

### 2.1. Fungi-Derived Enzymes

Among the most prominent options within this category, we can find AN-PEP (*Aspergillus niger* prolyl endopeptidase), a fungus-derived enzyme with activity at the stomach’s pH values and resistant to degradation by pepsin. Although this enzyme is already available on the market, conflicting studies exist on its efficacy. In initial studies, AN-PEP was shown to accelerate gluten digestion [11], to partially degrade gluten in a gastric simulator [12] and to significantly digest gluten in two clinical trials (NCT02060864 and NCT01335503) [13,14]. Nevertheless, in subsequent phase 1 and 2 pilot studies (NCT00810654) [15] and an exploratory phase 4 study (NCT04788797) [16], no difference in the presence of GIPs and other relevant biomarkers between the placebo and AN-PEP groups was found. The authors stated in a previous publication that “AN-PEP should not be used to replace a gluten free diet, but rather to support digestion of occasional and/or inadvertent gluten consumption”. To this end, current research around the AN-PEP enzyme is targeted at achieving gluten-free foodstuffs, especially bread [17,18], beer [19] and pizza [20].

Another recent fungi-derived enzyme aiming to treat CeD is AMYNOPEP, a combination of two polypeptides from *Trichophyton rubrum*, a leucine aminopeptidase 2 displaying aminopeptidase activity (AP) and a dipeptidylpeptidase IV (DPPIV) [21]. A study on AMYNOPEP reported full degradation (99.5%) of 33-mer into single and dipeptide amino acids in vitro when used in a 1:10 enzyme/33-mer ratio [22]. In healthy volunteers (German Register of Clinical Studies, DRKS00033099), 33-mer was administered alone or mimicking more complex meals through addition of apple juice, a whey protein drink or wheat gluten, with or without AMYNOPEP. Results from this proof-of-concept study showed faster and larger degradation of 33-mer when AMYNOPEP was administered [22].

Other fungi-derived enzymes are a DPPIV from *Aspergillus oryzae* or an aspergillopepsin from *Aspergillus niger* (*ASP*) [23]. While both DPPIV and ASP alone failed to efficiently cleave GIPs, a phase 1 and 2 clinical trial (NCT00962182) using a cocktail of both enzymes (STAN1) reported moderate gluten detoxification properties [23].

**Table 1:** Gluten-degrading enzymes.

Origin	Species	Name	Type	Phase, Clinical Trial, Sponsor	Ref.
Fungi	*A. niger*	AN-PEP	Serine prolyl endopeptidase	Interventional (NCT02060864, NCT01335503), Phase 1 and Phase 2 (NCT00810654), Phase 4 (NCT04788797)/DSM Food Specialties	[11,12,13,14,15,16]
	*A. niger* and *A. oryzae*	STAN1	Aspartate aspergillopepsin (ASP) + serine dipeptidyl-peptidase IV (DPPIV)	Phase 1 and phase 2 (NCT00962182)/Heim Pal Children’s Hospital	[23]
	*A. oryzae*	Flavourzyme	Mix of endo- and exo-peptidases	In vitro	[24]
	*T. rubrum*	AMYNOPEP	Leucine aminopeptidase 2 and DPPIV	German Register of Clinical Studies, DRKS00033099	[21,22]
Bacteria	*R. aeria* and *R. mucilaginosa*	*R. aeria* and *R. mucilaginosa*	Subtilisin-type serine endopeptidases	In vitro	[25]
	*A. sendaiensis*	Zamaglutenase/TAK-062 (from KumaMax and Kuma062)	Serine endopeptidase	Phase 1 (NCT03701555), Phase 2 (NCT05353985)/Takeda	[26,27,28]
	*B. gladioli*	Bga1903	Serine endopeptidase	In vitro	[29]
	*A. A8*	Endopeptidase 40 (E40)	Endopeptidase	In vitro	[30]
	*M. xanthus*	MX PEP	Prolyl endopeptidase	In vitro	[31]
	*F. meningosepticum*	FM PEP	Prolyl endopeptidase	In vitro	[32,33]
	*S. capsulate*	SC-PEP	Prolyl endoprotease	In vitro	[34]
	*B. licheniformis*	Alcalase	Serine endopeptidase	In vitro and in vivo	[24,35]
Plant- and food-derived enzymes	*N. ventrata*	Celiacase (neprosin)	Prolyl endopeptidase	In vitro and in vivo	[36,37]
	Pineapple (*A. comosus*)	Bromelain	Cysteine protease	In vitro	[38]
	Fig latex (*F. carica*)	Ficin	Cysteine protease	In vitro	[38]
	Hayward Kiwi *(A. deliciosa* cv. Hayward)	Actinidin	Cysteine protease	In vitro and in vivo	[39,40,41]
	Papaya latex (*C. papaya*)	Papain/Caricain (crude or purified)	Cysteine protease	In vitro and in vivo	[24,35,42]
	*T. aestivum*	Triticain-α	Cysteine protease	In vitro	[43]
	*Hordeym vulgare* (Barley)	EP-B2	Cysteine endoprotease	In vitro and in vivo	[32]
Combined (Bacteria + Plants)	*S.* *Capsulate* + *H. vulgare* (Barley)	Latiglutenase (ALV003 or IMX003)	Glutamine-specific cysteine peptidase + modified serine prolyl-specific oligopeptidase	Phase 1 (NCT00626184, NCT00669825), Phase 2 (NCT00959114, NCT01255696, NCT01917630, NCT03585478)/Immunogenics, LLC	[44]

### 2.2. Bacteria-Derived Enzymes

An early study identified around 60 bacteria naturally present in the oral cavity with gluten-hydrolyzing properties, from which *Rothia mucilaginosa* and *Rothia aeria* displayed the highest activity towards gluten degradation [25]. Later on, it was discovered that *R. aeria* achieves gluten detoxification through the BAV86562.1 subtilisin. In a preclinical study, addition of live or dead *R. aeria* to regular chow led to a 20–33% larger degradation of gliadins and immunogenic epitopes in vivo in BALB/c mice compared to regular chow [45].

Zamaglutenase (TAK-062, previously Kuma-062 or KumaMax) [26,27] is a serine endopeptidase derived and modified from the bacterial enzyme kumamolisin-As produced by *Alicyclobacillus sendaiensis*. Its main strength is that it targets the proline-glutamine (P-Q) motif instead of just one of either amino acid residues. It has proteolytic activity at a large pH range (2.5–6.0) and has been shown to degrade a 49.0–99.9% of the gluten present in a complex meal (1–6 g) 20–65 min after its intake. The phase 1 clinical trial (NCT03701555) also demonstrated its safety and tolerability at doses ranging from 100 to 900 mg [26]. A phase 2 trial (NCT05353985) has been completed but results have not been made available [28].

Bga1903 is a serine endopeptidase enzyme originating from *Burkholderia gladioli* [29]. In a previous study, it was reported to effectively degrade the 33- and 26-mer gluten-derived immunotoxic peptides in vitro and in beer in conjunction with peptidases from *A. niger*. While the mature version of Bga1903 is not considered to be ideal for gluten degradation, its optimum pH of 7.0 highly differs from the acidic conditions in the stomach, and the authors indicated that the enzyme could be modified in order to better perform as an oral therapy for CeD or so as to be targeted to reduce gluten content in foods.

Another bacterial-derived enzyme, Endoprotease 40 (E40), secreted by *Actinoallomurus* A8, was reported to be pepsin and trypsin resistant and activated at a pH ranging from 3 to 6 [30]. E40 achieved complete in vitro degradation of 33-mer and whole gliadin at 1:48 and 1:96 molar ratios, after 30 and 240 min, respectively, as assessed via high-performance liquid chromatography (HPLC). Moreover, E40-treated gliadin samples contained gluten levels below 20 ppm, when analyzed by an enzyme-linked immunosorbent assay (ELISA). Treatment of T cells obtained from CeD patients with TG2-deamidated E40-digested gliadin did not lead to the secretion of IFN-ɣ, contrary to what was observed for untreated samples.

Other enzymes which were researched in the past but are no longer being worked on include the bacterial prolyl endopeptidases from *Myxococcus xanthus* (MX PEP) [31] and *Flavobacterium meningosepticum* (FM PEP) [33,46].

### 2.3. Plant- and Food-Derived Enzymes

Neprosin is a prolyl endopeptidase naturally secreted by the carnivorous plant *Nepenthes* × *ventrata*. A previous study [37] showed that a combination of two proteolytic components from *N. ventrata*, nepenthesin and neprosin (in a 4:1 rate) displayed high gluten-degrading efficacy and prevented accumulation of IELs in vivo using HLA-DQ8 non-obese diabetic (NOD/DQ8) mice. Moreover, the proteolytic efficiency in vitro of the combined enzymes was found to be much higher than the readily available AN-PEP enzyme, thus requiring smaller amounts to achieve similar degradation rates. In a subsequent study [36], neprosin was shown to be capable of degrading gliadin in a 1:500–1000 enzyme/gliadin ratio. Moreover, it was able to efficiently function at the temperature and pH of the stomach both in vitro and in vivo, where it degraded the gluten administered to mice by up to 90%. Celiacase is a new gluten-degrading enzyme originating from Neprosin, which has been modified to achieve higher peptidolytic activity, as well as improved expression and stability [47].

Several studies have focused on enzymes naturally present in the fruit, latex or root from fruit trees. Two of them are Bromelain, an enzyme encompassing multiple cysteine proteases originating from pineapple (*Ananas comosus*), and ficin, a cysteine protease derived from the latex of the Ficus tree (*Ficus carica*). Digestion of gliadin and gliadin-derived 19-mer peptide by a mixture of bromelain and ficin (BF) led to significantly lower toxicity on Caco-2 cells and decreased intestinal permeability, similar to that of treatment with Larazotide acetate [38] (see Section 2.3. Intestinal permeability modulators).

Another food-derived enzyme is actinidin, a cysteine protease from Hayward kiwifruit (*Actinidia deliciosa* cv. Hayward), which showed small gluten degradation properties in rats, specifically for ω-gliadin [39], and a maximum degree of 15% gluten hydrolysis in a gastrointestinal simulator. Nevertheless, actinidin was the best-performing enzyme when compared to other naturally sourced enzymatic compounds like papain or bromelain [40,41].

A comparative study assessed the gluten-degrading capacity of three enzymes from natural origins: papain from the papaya-fruit latex (*Carica papaya*), flavourzyme from the fungus *Aspergillus oryzae* and alcalase from the bacterium *Bacillus licheniformis*, which were compared to animal trypsin, α-chymotrypsin and pepsin. The authors concluded that sequential treatment of wheat flour with alcalase and papain was the most effective combination, leading to the lowest immunoglobulin (Ig)E-binding [24]. In a subsequent study, BALB/c mice were sensitized with either regular gliadins, phosphorylated gliadins or alcalase- or papain-hydrolyzed gliadins. All pre-treatments significantly decreased serum total IgE, histamine, IFN-γ and IL-4 levels, compared to those in mice receiving untreated gliadin [35]. Another study obtained crude caricain/papain from papaya latex, purified it, and demonstrated that treatment of wholegrain bread fermented at 30–37 °C from 5 to 7 h with purified caricain/papain managed to reduce up to 98% of the gliadin originally present, and that the purified enzyme worked better than its crude version [42]. Another comparative study evaluated wheat-, rye- and barley-gluten degradation by either papain, bromelain, alcalase, flavourzyme, pepsin and trypsin [48]. The results showed that papain was capable of digesting gluten below the limit of detection for all food matrices, and the same was observed for alcalase-, flavourzyme- and pepsin-digested wheat and rye. The poorest-performing enzymes were bromelain and trypsin.

In terms of cereal-derived enzymes, triticain-α is a cysteine protease present in wheat (*Triticum aestivum*) [43] with potential gluten-hydrolyzing properties based on its cleavage sites and stability in acidic conditions. Nevertheless, these have not been proved in in vitro or in vivo experiments.

### 2.4. Combined Bacterial- and Plant-Derived Enzymes

Latiglutenase [44] (formerly ALV003 or IMX003) is composed of two glutenases active at the stomach’s pH in a 1:1 ratio, namely EP-B2, a cysteine endoprotease originating from germinating barley seeds [32], and SC-PEP, a prolyl endoprotease (PEP) from *Sphingomonas capsulate* able to cleave gluten at the proline residues [34]. Previous studies have shown that pre-treatment of gluten with this enzyme reduced the IFN-γ levels in blood but was not linked to a change in symptoms [44]. Two phase 1 (NCT00626184 and NCT00669825) and phase 2 (NCT00959114 and NCT01255696) clinical trials demonstrated that Latiglutenase was well tolerated at doses ranging from 100 to 1800 mg [49] and was able to prevent development of villous atrophy in patients following a GFD containing traces (2 g gluten per day) [50]. Nevertheless, in another phase 2 clinical trial (NCT01917630), Latiglutenase was not capable of reducing histologic and symptom scores in symptomatic CeD patients following a GFD [51]. In a more recent phase 2 trial (NCT03585478), it was demonstrated that Latiglutenase intake at a 1200 mg dose was capable of reducing the damage associated with daily intake of gluten traces (2 g/day) in CeD patients otherwise following a GFD, as observed by both a decrease in symptom- and histological-severity values [52]. While some results of the aforementioned trials are promising, others are conflicting, which may indicate that Latiglutenase could be an option to avoid histological damage derived from the presence of gluten traces in meals but would not be an alternative to a GFD.

### 2.5. Limitations and Strengths of Current Gluten-Degrading Enzymes

Strengths:

- Precise target: Gluten-degrading enzymes directly target the root problem in CeD, i.e., the GIPs that are resistant to digestion and then trigger immune responses. Their mechanism is rational and well defined, making them appealing therapeutic candidates.
- High safety and tolerability: Due to enzyme specificity, these are usually easily tolerated and do not interfere with other biological pathways.
- Oral delivery: Most researched enzymes are proposed to be delivered orally in a pill format before meals, which is easy and practical for patients.
- Role as a GFD-adjuvant: Some of the enzymes show potential in supporting digestion of inadvertent gluten exposure, which is a practical issue for patients strictly adhering to a GFD.

Limitations:

- Physiological barriers: Maintaining enzyme activity through the acidic, protease-rich environment of the stomach and achieving effective gluten degradation in real-meal contexts remains challenging, especially in mixed meals with complex matrices. From all the reviewed gluten-degrading enzymes, only a few have managed to overcome this frequent hindrance.
- High gliadin/enzyme ratio: Most of the enzymes developed required a high amount of the active proteins in order to properly degrade gluten in an effective manner.
- Site-specific enzyme activation: Effective gluten-degrading enzymes must be activated at the stomach’s acidic pH, where complete gliadin degradation should occur, and revert to an inactive state as pH rises along the gastrointestinal tract (GIT), as other ways GIPs may persist and trigger downstream immune responses.
- Inconsistent clinical efficacy: Despite promising in vitro and preclinical results, many enzymes have failed to show consistent benefit in human trials. Hence, since no enzyme therapy to date can serve as a standalone alternative to a GFD, even the most promising candidates are currently seen as adjunctive, not curative.

Due to their strengths and despite their limitations, which do not apply to all enzymes currently under investigation, gluten-degrading enzymes represent one of the most promising therapeutic approaches to CeD, given their high target specificity, safety, and ease of administration.

## 3. Gluten Direct Neutralization

Another strategy that has been proposed in the search for alternatives to a GFD is that of gluten neutralization, either through the use of targeted antibodies (Abs) or by means of complexing gluten to other molecules, thus rendering it unavailable to interact with the host’s immune system (Table 2). The main neutralization strategy consists of the IgY Ab from the egg yolk (AGY) of hens, which have been previously hyper-immunized against gliadin [53]. An important advantage of this method is that anti-gliadin IgY is not absorbed into the blood by humans; thus, it can act in the GIT while going undetected by the immune system. Moreover, it is easily producible in large amounts. A previous study showed in vivo with BALB/c mice that, after AGY-010 intake at a 1:5 AGY/gliadin ratio, only less than 1% of the gliadin administered reached the animal’s GIT [53]. Posteriorly, AGY-010 was reported to be safe for consumption in a phase 1 4-week clinical study (NCT01765647) [54] with biopsy-proven CeD patients following a GFD. A phase 2 (NCT03707730) [55] study has been completed but the results are not available yet.

Alternatively, BL-7010, a poly(hydroxyethyl methacrylate-co-styrene sulfonate) (P(HEMA-co-SS)) copolymer, was shown to effectively perform gliadin sequestration in both in vitro cell cultures and HLA-HCD4/DQ8 or NOD-DQ8 mice [56]. As a result of gliadin complexation with BL-7010 and the smaller amounts of GIPs consequently generated, the authors reported a decrease in cell mortality, IEL infiltration and paracellular permeability. BL-7010 also led to a higher villous height/crypt depth ratio (Vh:Cd) and was shown to be safe in rats at high doses [57]. While a phase 1 and 2 study was registered in 2013 (NCT01990885), its results have not been made available yet [58].

### Limitations and Strengths of Current Gluten Neutralization Treatments

Strengths:

- Early incidence: Since these strategies aim to block gluten passing through the epithelial layer, they act on the very first step of CeD pathophysiology, preventing the derived immune reaction before its initiation.
- Safety and tolerability: The anti-gliadin IgY antibodies from egg yolk are not absorbed systemically, significantly reducing the risk of immune reactions. This makes them particularly safe as oral therapies.
- Ease of production: Anti-gliadin IgY antibodies can be produced in large quantities at relatively low cost, offering a practical advantage over more complex biologics.
- Oral delivery: Both proposed treatments would be delivered orally, which generally show better acceptance scores by patients than other methods (e.g., vaccines).

Limitations:

- Uncertain site of action: It remains unclear where gluten neutralization primarily occurs, whether in the stomach or further along the GIT, which would allow for GIPs to be generated and absorbed into the lamina propria, thus triggering an immune reaction. Additionally, the stability and resistance of these agents to gastric pH remain insufficiently characterized.
- Limited clinical data: While preclinical studies show promise, robust efficacy data in humans are still lacking. The results of critical phase 2 trials are not yet published, creating uncertainty about real-world effectiveness.

## 4. Intestinal Permeability Modulators

Intestinal permeability (IP) is a common trait of CeD, although the causal effect is still under discussion: does having a “leaky gut” contribute to development of CeD, or does the overall inflammation characteristic of CeD lead to increased gut permeability? Despite what comes first, reducing the CeD-associated IP could not only help reduce the number of GIPs that cross from the intestinal lumen to the lamina propria, but it would also prevent microbial antigens and toxins from trespassing and causing inflammation [64]. To this end, multiple treatments have been researched, which aim to keep the tight junctions (TJs) between intestinal epithelial cells actually “tight” (Table 2).

One of the forerunners on this race is Larazotide acetate (previously AT-1001), a synthetic octapeptide that inhibits paracellular permeability through its function as a zonulin inhibitor [60]. Briefly, zonulin is a protein that modulates TJs and, in normal physiological conditions, it regulates their temporary opening to facilitate nutrient absorption and immune surveillance. Nevertheless, zonulin overexpression in CeD leads to chronic opening of the TJs [65]. Previous clinical studies have shown conflicting results; an initial study consisting of the administration of 2.5 g of gluten and 12 mg of Larazotide acetate for 7 days (*n* = 21) reported a prevention of IP (measured in lactulose-to-mannitol, LAMA ratios) and a decrease in both IFN-ɣ secretion and Gastrointestinal Symptoms Rating Scale (GSRS) values (NCT00362856, NCT00386165) [60]. A second trial (*n* = 86) testing four different treatment doses (0.25, 1, 4 or 8 mg) and a similar gluten intake of 2.4 g for 14 days reported no difference in the LAMA ratio or anti-tTG2 levels between the placebo and treatment groups but observed a better GSRS score in Larazotide vs. control patients undergoing a gluten challenge (NCT00362856) [61]. The same cohort was also used in a phase 1 study to evaluate Larazotide’s safety and tolerance (NCT00386490), which was deemed to be well tolerated. In a subsequent phase 2 cohort (NCT00492960 and NCT00889473), patients received 900 mg of gluten thrice per day, to a daily amount of 2.7 g of gluten, and 1, 4 or 8 mg of Larazotide 15 min before each meal for 6 weeks [62]. The authors reported a significant decrease in anti-tTG2 IgA levels in all the treatment groups, compared to the placebo one, but no differences in LAMA ratios. On a different direction, Larazotide acetate was tested in CeD patients (*n* = 342) following a GFD but with ongoing symptoms. Four doses were tested (0.5, 1 or 2 mg thrice daily) for 4 weeks and only the 0.5 mg group achieved the primary end point, i.e., a significant decrease in GSRS scores (NCT01396213) [59]. A 2019 phase 3 clinical trial (NCT03569007) was terminated without completion by the sponsor (9 Meters Biopharma, Inc.) [63].

Another recent IP regulator for the treatment of CeD is IMU-856, a modulator of sirtuin 6 (SIRT6) [66]. SIRT6 overexpression has been reported to display a protective effect against the loss of function by TJs and histopathological damage in a mouse model of colitis [67]. In a phase 1 clinical trial (ACTRN12620000901909, Australian New Zealand Clinical Trials Registry and CT-2020-CTN-01997-1, Therapeutic Goods Administration Clinical Trial Notification), IMU-856 administration at different doses alongside gluten was found to cause mild but frequent treatment-emergent adverse events (TEAEs). Moreover, no significant improvements in histology parameters because of IMU-856 administration, and regardless of dose, were found.

### Limitations and Strengths of Current Intestinal Permeability Modulators

Strengths:

- Disease target: The focus on intestinal permeability directly addresses a core feature of celiac pathophysiology, i.e., the disruption of TJs, which will then prevent access of GIPs into the lamina propria. Moreover, since these treatments typically act locally within the gut, systemic immune suppression and the risk of broader side effects common with immunomodulators are minimized.
- Safety and tolerability: Both existent strategies have demonstrated good tolerability in phase 1 and 2 trials, with mild or manageable adverse events, making them promising from a safety standpoint.
- Oral delivery: Both proposed treatments would be delivered orally, which generally show better acceptance scores by patients than other methods (e.g., vaccines).
- Symptom relief in some trials: Larazotide acetate has shown symptomatic benefit in several trials, even when histological or biomarker outcomes are inconsistent. This suggests potential value as an adjunct therapy for patients with persistent symptoms despite adherence to a GFD.

Limitations:

- Indirect activity: IP modulators do not alter gliadin itself; therefore, gliadin levels and GIP production remain unchanged. Their action is limited to preventing peptide translocation into the lamina propria, meaning that even minimal peptide crossing may still be sufficient to initiate CeD pathophysiology.
- Limited clinical effectiveness and inconsistent outcomes: No current IP modulator has demonstrated the ability to fully prevent villous atrophy or histological damage in response to gluten exposure, which is a key therapeutic goal in celiac disease. Moreover, while some studies reported reduced antibody levels or symptoms, others failed to demonstrate significant improvements in intestinal permeability or histological recovery. Results are often dose-dependent and inconsistent across studies.
- Uncertain causality in pathogenesis: It remains unclear whether increased IP is a cause or a consequence of CeD. This ambiguity complicates the rationale for prioritizing it as a standalone therapeutic target.

## 5. Immune Modulators

### 5.1. TG2 Inhibitors

As previously mentioned, the enzyme TG2 is one of the key players in the autoimmune reaction elicited by exposure to dietary gluten in CeD patients. Briefly, the GIPs that reach the lamina propria are deamidated by TG2, which significantly enhances its affinity to the HLA-DQ2/DQ8 receptors in APCs. Hence, TG2 inhibition is one of the most evident strategies to prevent CeD. To this end, ZED1227 (TAK-227), a selective oral TG2 inhibitor, was administered to CeD patients at one of three doses (10, 50 or 100 mg) alongside a 3 g gluten challenge for 6 weeks. Results of a phase 2 clinical trial (2017-00224-30, CEC-3 EudraCT number) showed that ZED1227 intake significantly decreased the extent of histological damage (measured as Vh:Cd), as compared to the placebo, but was not capable of completely preventing intestinal epithelial injury [68]. It was also deemed safe in two phase 1 trials (2014-003044-13 and 2015-005283-42) [69].

Similarly, another TG2 inhibitor, GSK-3915393, was being studied for CeD, but after phase 1 completion, the study was discontinued by the sponsor (NCT04604795) [70] and is currently only being studied for Idiopathic Pulmonary Fibrosis [71].

### 5.2. Anti-IL-15 Treatments

IL-15 has a crucial role in the pathogenesis of CeD. It has been shown that overexpression of IL-15 in the lamina propria and in intestinal epithelial cells (IECs) is essential for the development of villous atrophy in CeD [72]. Briefly, the conjunction of IL-15 and other pro-inflammatory cytokines such as IFN-ɣ promotes the acquisition of a CD8+ cytotoxic phenotype by IELs, which then mediate tissue damage after acquiring a killer phenotype. Moreover, it prevents the differentiation of regulatory T cells and promotes loss of oral tolerance [5,73]. Hence, targeting IL-15 is one of the strategies being investigated for treatment of CeD (Table 3). AMG 714 (or PRV-015) is an injection-administered IgG1κ-type human monoclonal antibody (moAb) against IL-15. It is hypothesized that it could inhibit the T-cell activation induced by IL-15 and the immune reaction that follows. AMG 714 has been studied in three phase 2 studies (NCT02637141, NCT02633020 and NCT04424927) for its efficacy and safety in adult patients with CeD [74], type II refractory CeD (RCeD-II) [75] and non-responsive CeD [76], respectively. Results from the first two trials showed no significant difference in the percentage of IELs, Vh/Cd, anti-TG2 or anti-DGP Abs between the treatment and placebo groups. In patients with non-responsive CeD, an improvement in diarrhea as measured by the Bristol stool scale was reported.

Another potential treatment targeting IL-15 inhibition is CALY-002, a moAb that neutralizes IL-15 through prevention of its interaction with IL-15Rβ [77], a receptor in IL-15-responder cells [78]. In a preclinical study regarding RCeD and eosinophilic esophagitis (EoE), the authors reported that treatment with CALY-002 significantly reduced the IELs in IL-15TgE mice (a mouse model of IL-15 overexpression) and the natural killer (NK) cells in cynomolgus monkeys. A downside of CALY-002 is that it does not prevent IL-15 recognition by receptor IL-15α. While CeD and EoE are not directly associated, they can co-occur [79]. A phase 1 study was recently completed (NCT04593251), but results are not available yet [80].

TEV-53408, an alternative anti-IL-15 moAb, managed to reduce the proliferation of mature NK cells, which have increased cytotoxicity and produce more pro-inflammatory cytokines [81]. A phase 1 study concluded that TEV-53408 was safe in healthy volunteers [82], and a phase 2 study was initiated in 2025 and is currently in the recruitment phase (NCT06807463) [83].

Alternatively, EQ102 is a gamma-chain receptor antagonist that inhibits both IL-15 and IL-21 [84]. Its sponsor company, Equillium, announced a phase 1 study of EQ102 in 2022 (ACTRN12622001449729) [85,86] and phase 2 study on EQ101 (IL-2, IL-9, and IL-15 inhibitor) and EQ102 in 2023 for Alopecia Areata (AA) and CeD (NCT05589610) [87]. While the results of this trial have not been made available to date, it was reported that EQ102 displayed lower bioavailability than expected. Hence, EQ302, an alternative treatment with increased potency, was developed from EQ102, its parent peptide [88]. As a result, work with EQ102 was terminated. Research on EQ302 is currently in the preclinical stage [89].

A recent study evaluated ex vivo the effect of aIL-15, an anti-IL-15 moAb, on the immune response responsible for intestinal epithelial damage from untreated CeD patients’ jejunal biopsies (Ethics committee of the San G. Moscati Hospital, Avellino, Italy, CECN/809) [90]. Results showed that those samples receiving aIL-15 displayed significantly smaller levels from molecules involved in tissue atrophy, including Fas, HLA-E, perforin and IFN-γ, compared to controls. While results are promising, further research on the effect of aIL-15 on relevant biomarkers of CeD, such as TG2 levels or intestinal epithelium histological measurements, is needed.

**Table 3:** Immune modulation strategies.

Strategy	Active Principle	Name	Phase, Clinical Trial	Sponsor	Ref.
TG2 inhibitors	SIRT6 modulator	IMU-856	Phase 1 (ACTRN12620000901909 or CT-2020-CTN-01997-1)	Immunic Australia Pty Ltd.	[66]
	TG2 inhibitor	ZED1227	Phase 1 (2014-003044-13) Phase 2 (2015-005283-42 and 2017-00224-30)	Dr. Falk Pharma GmbH	[68,69]
	TG2 inhibitor	GSK3915393	Phase 1–Discontinued (NCT04604795)	GlaxoSmithKline	[70]
IL-15 inhibitors	IL-15 inhibitor (moAb)	AMG 714 (PRV-015)	Phase 2 (NCT02633020, NCT02637141 and NCT04424927)	Amgen and Provention Bio (Sanofi)	[74,75,76,91]
	IL-15 inhibitor (moAb)	CALY-002	Phase 1 (NCT04593251)	Calypso Biotech BV	[77,80]
	IL-15 inhibitor (moAb)	TEV-53408	Phase 1 (NCT06807463)	Teva Branded Pharmaceutical Products R&D LLC	[82,83]
	IL-15 and IL-21 inhibitor (gamma chain receptor antagonist)	EQ102	Phase 1 and 2 (ACTRN12622001449729)	Equillium	[84,85,86,87,88]
	IL-15 inhibitor (moAb)	aIL-15	Ex vivo	ISA-CNR, ELFID *	[90]
Lymphocyte migration inhibitors	CCR9 antagonist	GSK1605786A/vercirnon (formerly CCX282-B)	Phase 2 (NCT00540657)	Amgen (formerly ChemoCentryx)	[92,93]
	α4β7 integrin inhibitor	PTG-100	Phase 1 (NCT04524221)	Nielsen Fernandez-Becker	[94,95]
HLA-DQ2 inhibitor	HLA-DQ2 inhibitor	Azidoproline	In vitro		[96,97]
JAK inhibition	Pan-JAK inhibitor	Tofacitinib (Xeljanz)	Phase 2 (EudraCT 2018-001678-10)	VU Medical Center	[98]
	JAK3 and TEC kinase family inhibitor	Ritlecitinib (LITFULO)	Phase 2 (NCT05636293)	Massachusetts General Hospital/Pfizer	[99,100,101]
CFTR potentiatior	CFTR potentiator	Ivacaftor (Kalydeco)	Case studies		[102,103,104]

* ISA-CNR = Institute of Food Science, National Research Council, ELFID = European Laboratory for the Investigation of Food-Induced Diseases.

### 5.3. Lymphocyte Migration Inhibitors

Lymphocyte migration plays a key role in CeD pathogenesis. Briefly, when GIPs enter the lamina propria and epitopes are taken up by APCs, lymphocytes migrate to the intestinal tissue, where they interact with APCs, are activated and initiate the gliadin-derived immune reaction. Current treatments focusing on the inhibition of lymphocyte migration in the context of CeD are available in Table 3. The CC motif chemokine receptor 9 (CCR9), primarily expressed in lymphocytes, plays an important role in CeD pathogenesis. Briefly, it promotes migration and homing of immune cells to the small intestine through interaction with the CC motif chemokine ligand 25 (CCL25, also known as TECK) receptor, highly expressed in both thymic and small intestine cells [105]. Previous studies have shown an increase in both CCR9 and CCL25 expression in CeD. Since, in the context of CeD, T cells in the gut contribute to damage of the epithelial tissue, it is hypothesized that any medication targeting the CCR9-CCL25 interaction could have a positive impact on reductions in intestinal inflammation. To this end, a phase 2 clinical study was undertaken to evaluate if CCX282-B, an antagonist of CCR9, could mitigate the effects of gluten ingestion in patients with CeD [92]. While a previous preclinical study in a mouse model of Crohn’s disease (TNF^∆ARE^) had shown that CCX282-B was capable of inhibiting the CCL25-induced chemotaxis mediated by CCR9A and B, and thus exert a preventive effect on intestinal inflammation [93], results in the context of CeD have not been published yet.

In a similar manner, PTG-100 is an inhibitor of the α4β7 integrin expressed in effector memory T cells, leading to their homing to inflamed areas in the GIT through interaction with mucosal addressin cell adhesion molecule 1 (MasCAM-1) [95]. Since a previous study showed a significant increase in MasCAM-1 expression in active CeD [106], blocking the α4β7 integrin/MasCAM-1 axis could be an effective strategy to prevent homing of effector T cells to the gut. While the results of a current phase 1 study on the safety and efficacy of PTG-100 in preventing gluten-induced inflammatory injury to the small intestine in patients with CeD have not been published yet (NCT04524221) [94], a previous clinical study in inflammatory bowel disease (IBS) showed an improvement in histological measurements [95].

### 5.4. HLA-DQ2 Inhibitors

Since the presence of the Class II HLA genes DQ2.2, DQ2.5 or DQ8 is essential for (but not indicative of) the development of CeD [107], one potential strategy for the prevention of this pathology is their inhibition, which would prevent the initiation of the ensuing immunological reaction. With this goal, one study substituted the proline residues in GIPs by azidoprolines, with the aim to use them to block the HLA-DQ2 molecules and prevent its interaction with regular GIPs [97]. Unfortunately, since the modified gluten peptides displayed similar binding affinity to HLA-DQ2 as normal gluten peptides, they could only partially prevent gluten binding to the receptors and the gluten-derived T-cell activation and immune reaction. Still, an encouraging observation was that the modified peptides complexed to HLA-DQ2 were not recognized by CD4+ T cells. In a following study, the authors actively looked for peptides with higher binding affinity to HLA-DQ2 than that of regular GIPs [96]. The peptide sequence ADAYDYESEELFAA displayed superior binding properties and was modified with non-natural peptides. Altogether, the newly developed sequences achieved a binding affinity that was up to 200-fold higher than that of gluten peptides. Nevertheless, after the last study concluded in 2010, this topic was not investigated further.

### 5.5. Janus Kinase (JAK) Inhibition

Intestinal permeability is considered a predisposing factor for the development of inflammatory diseases, including CeD. JAKs are a family of intracellular tyrosine kinases that are critical in cell signaling, especially in immune and blood cell functions [108]. The JAK-Signal Transducer and Activator of Transcription (STAT) pathway has a role in the regulation of IP through its selective activation or repression of genes involved in inflammation, cell proliferation, survival and differentiation. Specifically, in CeD, binding of cytokines such as IL-15 or IFN-γ to their corresponding receptors in epithelial cells can trigger the JAK-STAT pathway. Briefly, the JAK1 and JAK3 enzymes are activated and phosphorylated, and they then recruit and phosphorylate STAT proteins, which translocate into the nucleus and promote the expression of pore-forming proteins such as claudin-2, while decreasing the expression of barrier-sealing claudins and other promoters of intestinal integrity, such as occludin or zonula occludens-1 (ZO-1) [108]. Hence, some of the treatments being investigated for the prevention of CeD are JAK inhibitors (Table 3). Tofacitinib, a pan-JAK inhibitor, was subcutaneously delivered to T3^b^-hIL-15 transgenic (tg) mice in a 30 mg/(kg·day) dose for 42 days. The authors reported a decrease in CD8+NKG2D+ T cells in blood and the intestinal mucosa, reduction in IELs and prevention of villous atrophy. Nevertheless, a significant increase in visceral adiposity was also noted [109]. While many clinical studies have been conducted for Tofacitinib and a wide array of pathologies, the first phase 2 study to assess its effect on CeD, and more specifically RCeD-II, was developed in 2019 (EudraCT 2018-001678-10) [98]. Patients received 10 mg twice daily for 12 weeks, and their symptoms (diarrhea, abdominal pain, weight loss) rapidly improved but reappeared on discontinuation of treatment. Nevertheless, a significant decrease in IELs was not observed, and mucosal improvement was reported for only half of the patients. Moreover, TEAEs were reported for all patients. Limitations of this study include the low patient numbers (*n* = 4) and no placebo group. Previous case studies on tofacitinib have reported histologic and serologic remission of CeD in a gluten-containing diet (GCD) [110], significant improvement in CeD-related dermatitis herpetiformis (DH) after failed response to first-line treatment (dapsone) [111] and CeD and microscopic colitis clinical and histological remission with and without a GFD [112].

Simultaneously, Ritlecitinib is a JAK3 and TEC (tyrosine kinase expressed in hepatocellular carcinoma) kinase family inhibitor [99]. Its use was approved in the USA and Japan in 2023 for the treatment of severe AA and has long been under investigation for vitiligo, ulcerative colitis and Crohn’s disease. Just recently, a phase 2 clinical trial was developed to evaluate the potential effects of Ritlecitinib in the context of CeD (NCT05636293) [100], which is still in the recruitment phase.

### 5.6. Cystic Fibrosis Transmembrane Conductance Regulator (CFTR) Promoter

CFTR is a protein that functions as a chloride channel on the surface of epithelial cells and, at the intestinal level, it is linked to the regulation of fluid secretion, maintenance of proper mucosal barrier function and protection against pathogens and inflammation. A previous study observed that the P31-43 α-gliadin 13-mer peptide (LGQQQPFPPQQPY) inhibits the function of the CFTR, which leads to epithelial stress, TG2 and IL-15 production, among others [104]. Ivacaftor (Table 3) is a CFTR potentiator commercialized for cystic fibrosis (CF) treatment, which, based on previous findings, could have a therapeutical effect on CeD [113]. While there is no ongoing trial on this topic, a few case studies from CF patients diagnosed with CeD and treated with Ivacaftor reported that an improvement in GI symptoms was only observed after adherence to a GFD and not drug related [102,103].

### 5.7. Limitations and Strengths of Current Immune Modulators

Strengths:

- Disease target: Immune modulators aim to intervene directly in the autoimmune cascade triggered by gluten exposure, including T-cell activation, cytokine release, and tissue damage. By targeting elements like IL-15, TG2, JAK-STAT signaling, or HLA-DQ2 binding, these therapies address the core immunopathology of CeD rather than just symptoms.
- Promising early results in specific areas: ZED1227 (TG2 inhibitor) has shown histological improvement in phase 2 trials, marking one of the most promising outcomes in disease-modifying therapy. Tofacitinib and Ritlecitinib (JAK inhibitors) showed a clinical improvement in RCeD-II, suggesting utility in hard-to-treat CeD. AMG 714 provided symptom relief in some cohorts, particularly for patients with non-responsive CeD.
- Mode of delivery: Most treatments are designed to be delivered orally.

Limitations:

- Safety and tolerability: Tofacitinib was associated with increased visceral adiposity and TEAEs in all patients studied. Long-term safety data for JAK inhibitors and systemic moAbs are still limited, particularly regarding immune suppression and infection risk.
- Mode of delivery: While some treatments are designed to be delivered orally, others, mainly the IL-15 inhibitors, should be administered through an injection, which may decrease patient acceptability.
- Site of action: Most immune modulators act downstream, after gliadin peptides have crossed the epithelial barrier and entered the lamina propria, where they can already trigger the autoimmune cascade. This timing allows for disease activation before therapeutic intervention can occur.
- Symptom relief without mucosal healing: Several immune modulators improved subjective symptoms (e.g., diarrhea, abdominal pain) but failed to correlate with objective markers like IEL counts or villous architecture.

## 6. Gluten Tolerization

Since exposure to dietary gluten is essential for the development of CeD, many strategies have focused on achieving immune tolerance to GIPs (Table 4). One such approach is Nexvax2, a vaccine whose main active principles are three 15/16-aminoacid-long peptides containing five immunodominant epitopes that interact with the HLA-DQ2.5+ receptor in CD4+ T cells [114,115]. While this treatment was considered safe after various phase I clinical trials (NCT00879749, NCT02528799 and NCT03543540) [114,115,116], a phase 2 study (NCT03644069) [117] concluded that after administration of escalating doses (from 1 µg to 750 µg for 5 weeks and then 900 µg for 11 weeks), Nexvax2 was not capable of reducing the symptoms associated with gluten consumption in CeD in a posterior 10 g gluten challenge at week 14. As a result, the study was terminated by the authors [118].

Another therapy under investigation is TAK-101 (or TIMP-GLIA), consisting of negatively charged gliadin-encapsulating nanoparticles [119]. In a phase 1 (NCT03486990) and phase 2 study (NCT03738475), patients following a GFD were administered ascending doses of TAK-101 (0.1–8 mg/kg up to 650 mg) through two 30 min intravenous infusions on days 1 and 8 within the 2 weeks prior to the start of the challenge and were then subjected to a 14-day oral gluten challenge (12 g/day for 3 days and 6 g/day the remaining time). Results from both trials concluded that TAK-101 was capable of reducing 88% of gliadin-specific IFN-γ-producing cells, and it also significantly prevented the degree of villous atrophy, among other indicators. Moreover, no serious TEAEs were reported. An additional phase 2 clinical trial with alternative doses/dosing strategies is currently under development, but the results are not available yet [120].

KAN-101 consists of a conjugation between a liver-targeting glycosylation signature and an α-gliadin deamidated peptide (KAN0009). It aims to induce gluten tolerance through the promotion of tolerogenic pathways, which then prevent T-cell activation after gluten exposure. In a phase 1 trial (NCT04248855) [121], KAN-101 was administered thrice intravenously in single ascending doses ranging from 0.15 to 1.5 mg/kg, followed by a gluten challenge of 9 g/day for 3 days. During the study, 67–79% of participants receiving KAN-101 reported TEAEs of mild to moderate severity. All doses significantly decreased plasma IL-2 after gluten exposure compared to the placebo group. While a subsequent phase 2 study was terminated by the sponsor (NCT05574010) [122], another phase 2 study, from which results are unavailable, was developed and recently completed (NCT06001177) [123].

Another potential treatment for CeD is TPM502, consisting of nanoparticles coupled with CeD-derived relevant antigens [124]. Although results of the phase 2 clinical trial have not been published yet (NCT05660109) [125], the project sponsor communicated in a press release that the drug was safe and that a decrease in T-cell-derived IL-2 and IFN-γ was observed at the highest dose. A total of 38 patients received two doses of TPM502 (ranging from 0.72 to 7.2 μmol) on days 1 and 15 and were subjected to a gluten challenge (6 g gluten) 7 days after the last infusion [124].

### Limitations and Strengths of Current Gluten Tolerization Strategies

Strengths:

- Disease target: These therapies aim to reprogram the immune system to tolerate gluten by directly targeting antigen-specific T-cell responses, rather than broadly suppressing immunity. This makes them highly specific and theoretically curative, addressing the disease’s underlying cause.
- Promising early results in specific areas: Agents like TAK-101 and KAN-101 have shown clear immunologic effects in clinical trials, including reductions in gliadin-specific IFN-γ-producing T cells, decreased IL-2 levels following gluten challenge and prevention of or reduction in villous atrophy.
- Safety and tolerability: Most candidates, including TAK-101, KAN-101, TPM502, and Nexvax2, demonstrated acceptable safety profiles, with only mild to moderate TEAEs and no serious safety concerns reported.

Limitations:

- Mode of delivery: Most agents are currently delivered intravenously (e.g., TAK-101, KAN-101, TPM502), which may limit patient acceptability and clinical practicality for long-term treatment or maintenance use.
- Limited clinical effectiveness and inconsistent outcomes: Nexvax2, despite strong immunological rationale and early safety, failed to reduce symptoms during gluten challenge in a phase 2 trial, so it was discontinued. Results of multiple phase 2 trials have not been made available.

**Table 4:** Gluten tolerization strategies.

Strategy	Active Principle	Name	Phase, Clinical Trial	Sponsor	Ref.
Gluten tolerization	Vaccine containing 5 immunodominant gluten epitopes	Nexvax2	Phase 1 (NCT00879749, NCT02528799, NCT03543540) Phase 2–terminated (NCT03644069)	Nexpep Pty Ltd. and ImmusanT, Inc.	[114,115,116,117]
	Gliadin-encapsulated nanoparticles	TAK-101 or TIMP-GLIA	Phase 1 (NCT03486990), Phase 2 (NCT03738475 and NCT04530123)	Takeda	[119,120,126]
	Liver-targeting glycosylation signature conjugated to an α-gliadin deamidated peptide	KAN-101	Phase 1 (NCT04248855, NCT05574010), Phase 2 (NCT05574010 and NCT06001177)	Kanyos Bio, Inc. (Anokion SA)	[121,122,123]
	Nanoparticles coupled with CeD-derived relevant antigens	TPM502	Phase 2 (NCT05660109)	Topas Therapeutics GmbH	[124,125]

## 7. Probiotics, Prebiotics, Synbiotics and Postbiotics

With CeD being a chronic enteropathy, it is not surprising that the gut microbiota plays a big role in its pathophysiology. It is widely recognized that most patients with CeD suffer from gut dysbiosis, including IP, inflammation of the intestinal epithelium and many gut-related symptoms such as diarrhea, bloating or constipation, even when on a GFD [127]. While more research is still needed to truly understand the numerous pathways in which the gut microbiota influences CeD pathogenesis, some of them have already been elucidated. A healthy intestinal microbiota produces beneficial metabolites crucial in overall body health, such as short-chain fatty acids (SCFAs) or mucous, which are known to help prevent IP [128]. Studies have also shown that the gut microbiota can exert immunomodulatory functions, like an increase in proliferation of IECs, in the production of antimicrobial peptides or regulation of the inflammatory response triggered by Toll-like receptors (TLRs) [128,129]. Moreover, functions of specific taxa have also been uncovered. Several studies have reported that, in CeD, a reduction in the abundance of several bacterial groups such as *Bifidobacterium*, *Lactobacillus* or *B. longum* and an increase in *Bacteroides*, *Staphylococcus*, enterobacteria, *B. vulgatus* or *E. coli* have frequently been found, when compared to control subjects [130,131,132,133]. It has also been reported that patients with active CeD with or without symptoms, with DH or with recurring symptoms after initiation of a GFD, present clearly differentiated gut microbiota profiles [134,135]. Because of the observed decrease in *Bifidobacterium* and *Lactobacillus*, and due to their recognized beneficial effects, many interventional studies target them, as well as other probiotics (live microorganisms that, when administered in adequate amounts, confer a health benefit on the host) [136]. Simultaneously, a lot of research is now focused on prebiotics (a substrate which is selectively utilized by host microorganisms conferring a health benefit), synbiotics (a combination of prebiotics and probiotics) and postbiotics (preparation of inanimate microorganisms and/or their components that confers a health benefit on the host) [136]. Nevertheless, only one study on prebiotics, and none for synbiotics and postbiotics, has focused on the potential effects of these compounds in CeD pathogenesis. The current existing research on pre- and probiotics in relation to CeD is summarized in Table 5.

### 7.1. Bifidobacterium

One candidate probiotic for CeD is *Bifidobacterium infantis* natren life start super strain (NLS-SS or Lifestart 2). In an initial interventional clinical trial (NCT01257620) [137], patients received 2 × 10^9^ colony-forming units (CFU)/capsule twice a day alongside 12 g of gluten daily. Results of the study suggested that NLS-SS was safe for consumption, but it did not achieve a significant decrease in serological markers. Even so, GSRS scores slightly improved. In a posterior phase 2 clinical study (NCT03271138) [138], the effect of NLS-SS on CeD patients on a GFD with persistent symptomatology was evaluated. Results showed a decrease in CeD symptoms and differential β-diversity of the stool microbiota between treatment and placebo groups. The presence of GIPs in fecal and urine samples did not vary between groups.

Within the *Bifidobacterium* genus, the probiotic *B. breve* BR03 and B632 strains have also been investigated in the context of CeD. As a result of a phase 1 and 2 clinical trial (NCT02244047) [139] with children on a GFD receiving 2 × 10^9^ CFU/daily for 3 months, a significant decrease in serum TNF-α levels was reported, which increased back on treatment discontinuation. The authors also observed a decrease in the *Firmicutes*/*Bacteroidetes* ratio and in *Actinobacteria,* which were normalized after probiotic intake [140,141].

*B. longum* CECT 7347 or ES1 is another strain that has been widely researched. An initial preclinical study used an animal model of gliadin-induced enteropathy and reported that CECT 7347 administration alongside gluten prevented cellular infiltration, caused a reduction in villi height and width [142] and led to an improvement in gliadin-derived iron deficiency in the liver [143]. Subsequently, an exploratory study with children on a GFD was undertaken [144]. Patients received 10^9^ CFU/daily for 3 months and, as a result, a greater height percentile, decreased peripheral CD3+ T-cell levels and less secretory IgA in stools were achieved. A pilot study was then conducted on non-celiac gluten sensitivity (NCGS) (Maggiore Polyclinic Hospital, study registration number: 1370) [145], where a decrease in all gluten-associated symptoms, except for anxiety, was reported after coadministration of ES1 alongside a GFD. In 2016, another study to assess the effect of ES1 on NCGS was registered (NCT02810301), but its status is unknown [146]. Two recent interventional clinical trials have evaluated the effects of *B. longum* CECT 7347 in healthy adults with mild to moderate digestive symptoms (NCT05367427) [147] or in patients with diarrhea-predominant IBS (IBS-D) (NCT05339243) [148]. In IBS-D, a decrease in symptom severity and an improvement in stool consistency after ES1 intake were reported.

Along another line, a previous study demonstrated that supplementation with a serine protease inhibitor (Srp) helped decrease gluten-related immunopathology [149]. A study with sensitized NOD/DQ8 tg mice demonstrated that administration of 10^9^ CFU of *B. longum* NCC2705, which produces a serine protease inhibitor, once a week during a 3-week period, partially prevented IEL infiltration and villi atrophy [150,151]. An interventional trial was started in 2018 for NCGS consisting of administration of two capsules twice daily for four days followed by a 3 g gluten challenge, but the results are not available yet [151].

In an in vitro study using Caco-2 cells, the ability of specific strains of *L. fermentum* and *B. lactis* to prevent intestinal damage caused by GIPs was evaluated [152]. The results showed that *B. lactis* decreased gliadin-induced intestinal permeability and reinforced TJs, as observed by a higher ZO-1 expression.

### 7.2. Lactobacillus

A probiotic mix composed of *Lactobacillus plantarum* HEAL9 and *L. paracasei* 8700:2 was evaluated in an interventional clinical study (NCT03176095) [153,154,155]. The results showed that probiotic administration at 10^10^ CFU/day for six months did not cause a significant change in tTG2-IgA or –IgG levels between groups, but a decrease in both NK and NKT cells was observed in the probiotic group. A subsequent interventional study on these probiotics is active but not recruiting (NCT04014660) [156].

While no other clinical trials have been undertaken for *Lactobacillus* and CeD, one study did evaluate the gluten-degrading capacity from Lactobacilli obtained from the small intestine of gluten-fed pigs [157]. Among them, *L. ruminis*, *L. johnsonii*, *L. amylovorus*, and *L. salivarius* showed the highest gluten degradation activities. Another study researched *L. brevis* KT16-2 and reported that it displayed in vitro gluten-hydrolyzing abilities [158]. Several other studies have focused on the potential beneficial effects of various *Lactobacillus* sp. in CeD, including *L. casei* ATCC 9595, *L. plantarum ITM21B*, *L. paracasei IMPC2.1* and *L. fermentum*, as well as *Bifidobacterium* lactis [159,160,161]. Probiotic administration (10^10^ CFU/4 times a week) to HLA-DQ8 tg gluten-sensitized mice led to an enhanced CD4+ T-cell response, as seen by increased IFN-ɣ and TNF-α expression. Later on, using the same mouse model with addition of cyclo-oxygenase inhibition, the administration of *L. casei* ATCC 9595 induced recovery of gliadin-derived villi atrophy and a decrease in TNF-α but not IL-2, levels [160].

### 7.3. Others

A previous study obtained and characterized gluten-hydrolyzing bacteria from wheat sourdough and curd. The *Bacillus* sp. GS 33, 143, 181 and 188 demonstrated survival properties at pH 2 and in the presence of 0.3% bile acids [162,163]. Similarly, another work identified four *Enterococcus faecalis* strains, as well as *Enterococcus mundtii, Bacillus cereus*, *Bacillus megaterium* and *Wickerhamomyces anomalus* strains present in fermented sourdoughs, which displayed gluten-hydrolyzing properties [164]. Among these, only *E. mundtii* QAUSD01 and *W. anomalus* QAUWA03 showed sufficient pH and bile salt tolerance to be considered for future research as probiotics in CeD.

A novel strain belonging to the *Bacteroides vulgatus* species (20220303-A2) and present in healthy but not in CeD subjects was isolated. Using a human gut organoid, the authors observed that *B. vulgatus* 20220303-A2 mitigated the increased cell death, IP and levels of inflammatory cytokines caused by gliadin exposure [165].

**Table 5:** Probiotic and prebiotic treatments.

Active Principle	Name	Phase, Clinical Trial	Sponsor	Ref.
*Bifidobacteria*	*B. infantis* NLS-SS	Interventional (NCT01257620) Phase 2 (NCT03271138)	Bai, Julio M.D. and Global Institute of Probiotics	[137,138]
	*B. breve* BR03 and B632	Phase 1 and 2 (NCT02244047)	University Medical Centre Maribor	[139,140,141]
	*B. longum* CECT 7347 (ES1)	Interventional (NCT05339243 and NCT05367427) Phase 2–unknow status (NCT02810301), Maggiore Polyclinic Hospital clinical trial 1370	Vedic Lifesciences Pvt. Ltd., Instituto de Investigación Hospital Universitario La Paz, Maggiore Polyclinic Hospital clinical, Exzell Pharma Inc.	[142,143,144,145,146,147,148,166]
	*B. longum* NCC2705	Interventional (NCT03775499)	Société des Produits Nestlé (SPN)	[149,150]
	*B. lactis*	In vitro and in vivo		[152,161]
*Lactobacillus*	*L. plantarum* HEAL9 and *L. paracasei* 8700:2	Interventional (NCT03176095 and NCT04014660)	Lund University	[153,154,155]
	*L. brevis* KT16-2	In vitro		[158]
	*L. casei* ATCC 9595	In vitro and in vivo		[159,160,161]
	*L. plantarum ITM21B*, *L. paracasei IMPC2.1*, *L. fermentum,*	In vitro and in vivo		[161]
*Bacillus*	*Bacillus* sp. GS 33, 143, 181 and 188	In vitro		[162,163]
	*B. vulgatus* 20220303-A2	Ex vivo		[165]
Multispecies probiotics	VSL#3	Phase 2 (ACTRN12610000630011)	Metametrix Clinical Laboratory, Diagnostic Insight and Sigma Pharmaceuticals Pty	[167,168]
	*Multispecies probiotic*	Interventional (NCT01699191)	University of Bari	[169]
	*Pentabioc* *el*	Interventional (NCT03857360)	Università Politecnica delle Marche	[170]
	P1: *B. breve* B632 and BR03, P2: *L. plantarum* LP14, *L. casei* subsp. *paracasei* LPC09, *L. rhamnosus* LR04	In vitro and in vivo	Pobiotical SpA	[171]
Bacteria & yeast	*E. mundtii* QAUSD01 and *W. anomalus* QAUWA03	In vitro		[164]
Yeast	*S. boulardii KK1*	In vivo		[172]
Helminth	*N. americanus*	Phase 1 (NCT02754609), Phase 1 and 2 (NCT01661933) Phase 2 (NCT00671138	Princess Alexandra Hospital, The Prince Charles Hospital and James Cook University	[173,174,175]
Prebiotics and Postbiotics	*Synergy 1* (Oligofructose-enriched inulin)	Interventional (NCT03064997)	Polish Academy of Sciences	[176,177,178,179]
	*Heat-treated B. longum* CECT 7347 (HI-ES1)	Interventional (NCT05339243 and NCT05367427) Phase 2–unknow status (NCT02810301), Maggiore Polyclinic Hospital clinical trial 1370	Vedic Lifesciences Pvt. Ltd., Instituto de Investigación Hospital Universitario La Paz, Maggiore Polyclinic Hospital clinical, Exzell Pharma Inc.	[142,143,144,145,146,147,148,166]

### 7.4. Multispecies Probiotics

The VSL#3 probiotic formulation, containing *Streptococcus thermophilus*, *L. plantarum*, *L. acidophilus*, *L. casei*, *L. delbrueckii* sp. *bulgaricus*, *Bifidobacterium breve*, *B. longum* and *B. infantis*, demonstrated a gluten-degrading capacity during a long fermentation of wheat flour. Moreover, addition of VSL#3-digested gliadins achieved smaller zonulin production by rat intestinal epithelial cells (IEC-6), compared to that of regular gliadin, and smaller CeD3+ cell infiltration in the mucosa of biopsies from CeD patients. Nevertheless, in BALB/c mice, VSL#3-digested gliadins were not capable of preventing the reduction in tissue transepithelial electrical resistance (TEER) caused by gliadin, thus leading to increased IP [168]. While many clinical trials have been developed for VSL#3 and a wide array of diseases, including IBS or Asthma, only one has researched its potential effect on CeD pathogenesis (ACTRN12610000630011) [167], and it concluded that VSL#3 intake did not cause a significant change in the fecal microbiota of CeD patients.

A few multispecies probiotics have also been researched for their effects on CeD. A probiotic combination containing two *Lactobacillus* and three *Bifidobacterium* sp. (*L. casei* LMG 101/37 P-17504, *L. plantarum* CECT 4528, *B. animalis* subsp. *lactis* Bi1 LMG P-17502, *B. breve* Bbr8 LMG P-17501, *B. breve* Bl10 LMG P-17500) was administered in a clinical trial (NCT01699191) at concentrations ranging from 5 to 10 × 10^9^ CFU/day, for 6 weeks in patients with CeD and IBS, while following a GFD. The results reported a decrease in GSRS scores [169]. The same authors had previously investigated the peptidase activity of 18 commercial probiotic lactobacilli strains (*L. casei* BGP93; *L. delbrueckii* subsp. *bulgaricus* SP5; *L. paracasei* LPC01 and BGP2; and *L. plantarum* BGP12, LP27, LP35, LP40, LP47, and SP1), reporting that pooling of 10 of these strains was capable of completely degrading all the GIPs present in a sample using a gastrointestinal simulator [180]. Moreover, when wheat bread was pre-digested with the same probiotic mix and applied to biopsies from CeD patients, IL-2, IL-10 and IFN-ɣ levels remained at baseline.

Another probiotic cocktail named Pentabiocel and composed of *L. paracasei* 101/37 LMG P-17504, *L. plantarum* 14D CECT 4528, *Bifidobacterium animalis* subsp. *lactis* Bi1 LMG P-17502, *B. breve* Bbr8 LMG P-17501 and *B. breve* BL10 LMG P-17500 was evaluated in a clinical trial (NCT03857360) [170]. Children newly diagnosed with CeD took the probiotic for 12 weeks, but no significant differences between the treatment and placebo groups in terms of anti-TG2 IgA Abs, fecal calprotectin or hemoglobin were found. Only the BMI Z-score was significantly higher in the probiotic group.

Two different probiotic combinations from the company Probiotical SpA were assayed both in vitro and in vivo in gliadin-sensitized BALB/c mice [171], showing promising results. Both P1 and P2 formulations, containing ~10^6^ active fluorescent units (AFUs) of *B. breve* B632 and BR03 (P1) and *L. plantarum* LP14, *L. casei* subsp. *paracasei* LPC09 and *L. rhamnosus* LR04 (P2), were administered daily to mice, along with gliadin. The results showed that probiotic administration significantly decreased TG2 expression and the levels of inflammatory cytokines in the small intestine down to baseline levels. It also achieved restoration of intestinal morphology and gliadin-mediated IP, as observed by decreased claudin-2 and -15 and increased occludin levels.

### 7.5. Yeast

In a gluten-sensitized BALB/c mouse model, administration of 10^8^ CFU/day *Saccharomyces boulardii* KK1 for 7 days alongside gluten partially prevented villi atrophy, as measured using the Vh/Cd ratio, IEL infiltration and both IFN-ɣ and TNF-α production. Nevertheless, it was not capable of decreasing IL-15 and TG2 production. The same study assayed joined administration of *L. paracasei* DC205 and DC412, which did not display the same preventive effect [172].

### 7.6. Helminth

Previous studies have shown that helminth infection can attenuate the severity of autoimmune and inflammatory diseases, such as autoimmune liver disease [181], IBS [182], colitis [183,184], multiple sclerosis [185] or allergic rhinitis [186]. Although the exact mechanism for these effects is not very well known, it is hypothesized they could exert beneficial effects through modification of the intestinal microbiota [175]. In order to elucidate the potential role of a helminth infection in the amelioration of CeD, a phase 2 clinical study (NCT00671138) was undertaken consisting of the infection of 20 CeD patients with the hookworm *Necator americanus* [174]. Unfortunately, the results showed no improvement in the pathology. In a subsequent phase 1 and 2 study (NCT01661933) [175], infection with *N. americanus* in CeD patients followed by a gluten challenge (10–50 mg/day) was associated with increased abundance of species belonging to the Bacteroides phylum and with an increase in microbiota richness and diversity. Although biopsies were collected, the study did not report data on gluten-related biomarkers such as villous atrophy. An ensuing phase 1 study (NCT02754609) reported similar results to the initial one, where infection with the hookworm did not have a positive impact on reducing gluten-associated symptomatology in CeD patients but also decreased their quality of life [173].

### 7.7. Prebiotics, Synbiotics and Postbiotics

An untapped research area in regard to CeD involves prebiotics, symbiotics and postbiotics. Since all of them target the intestinal microbiota, and it is acknowledged that a lot of investigation is still needed to better understand the specific, and thus modifiable, pathways in which the gut microbiota prevents, promotes or alleviates CeD, it is only logical that very few projects have been targeted to elucidate the role of pre-, syn- and postbiotics on CeD pathogenesis. Currently, there are only two projects researching the effect of prebiotic intake on CeD. As mentioned before, decreased amounts of *Lactobacillus* and *Bifidobacterium* are commonly found in both active and treated CeD. To this end, the goal of one study was to increase such beneficial bacteria through the administration of the oligofructose-enriched inulin probiotic Sinergy 1. A clinical trial was conducted in CeD children following a GFD (NCT03064997) [177], which consisted of 1 intake/day of a 10 g Synergy 1 dose for 3 months. Results from the study showed that prebiotic supplementation did not cause side effects and significantly increased *Bifidobacterium*, acetate and butyrate fecal levels [179] and glutamine plasma concentration [178]. Conversely, Synergy 1 did not increase barrier integrity, with the limitation that most study subjects had normal IP values at study initiation [187]. Moreover, a decrease in serum hepcidin, which is involved in intestinal iron absorption, was observed [176].

Simultaneously, heat-treated B. longum CECT 7347 (HT-ES1) has also been researched in two clinical trials (while compared to its active counterpart) focusing on IBS (NCT05339243) [148] and healthy adults with moderate gastrointestinal symptoms (NCT05367427) [147]. For the latter, HT-ES1 intake did not have an effect on GSRS scores, but it caused a decrease in total and non-high-density lipoprotein (HDL) cholesterol and an increase in the butyrate-producing *Faecalibacterium* genera. In IBS, both an improvement in stool consistency and a decrease in symptom severity were reported as a result of HT-ES1 intake.

### 7.8. Fecal Microbiota Transplantation (FMT)

Along the same line as all aforementioned strategies, FMT aims to modify the intestinal microbial composition to prevent dysbiosis and its associated detrimental effects. While not many studies have aimed to uncover the potential role of FMT in CeD pathogenesis, one case study did report that, after performing FMT on a patient with RCeD-II due to persistent *Clostridium difficile* infection (CDI), remission of CeD symptoms and recovery of intestinal morphology were observed [188]. While FMT is currently only recommended for CDI, an existing clinical trial (NCT04014413) is currently recruiting patients with intestinal diseases, including CeD, to evaluate the safety and efficacy of FMT [189].

### 7.9. Limitations and Strengths of Current Probiotic, Prebiotic, Synbiotic and Postbiotic Strategies

Strengths:

- Disease target: CeD is increasingly recognized as a condition involving gut dysbiosis, even in patients adhering to a GFD. The microbial imbalance is associated with inflammation, increased IP and persistent symptoms, making microbiota-targeted therapies highly relevant.
- Uncertain mechanisms of action: A key limitation of probiotic use in CeD is the lack of clear evidence regarding the exact mechanism of action. While some studies suggest potential benefits, the specific pathways through which probiotics exert these effects in the context of gluten-induced autoimmunity remain largely speculative. This uncertainty hampers the development of targeted formulations and limits confidence in their therapeutic efficacy.
- Broad immunomodulatory potential: Certain strains (e.g., *B. breve*, *B. longum*, *L. casei*) have shown abilities to decrease pro-inflammatory cytokines, improve intestinal barrier function, modulate immune cell populations or increase beneficial metabolites like SCFA.
- Symptom relief in some studies: Several probiotic combinations (e.g., *B. infantis* NLS-SS, ES1 or some multispecies formulations) showed improvements in GI symptoms, including bloating, stool consistency, and GSRS scores, especially in patients on a GFD with persistent symptomatology.
- Safety and tolerability: Across most trials, no serious TEAEs were reported. Even novel combinations or postbiotics (e.g., heat-treated ES1) were well tolerated, including in children.
- Complementary to existing therapies: Generally, these strategies do not aim to replace a GFD but rather enhance its effectiveness by improving symptom control, supporting mucosal healing, and addressing non-responsive or partially responsive CeD.
- Mode of delivery: Most treatments are designed to be delivered orally.

Limitations:

- Limited clinical efficacy and inconsistent outcomes: Many interventions showed no significant changes in serological markers, histological damage or IP. Some promising in vitro or in vivo studies failed to translate into measurable clinical benefits in humans (e.g., VSL#3, Pentabiocel, *S. boulardii*).
- Uncertain causality in pathogenesis: Similar to intestinal permeability modulators, it remains unclear whether increased gut dysbiosis is a cause or a consequence of CeD. Hence, the extent of the effect of improvement in the gut microbiota profile in CeD symptomatology and disease biomarkers is unclear.
- Few rigorous or large-scale trials: Most trials were small, exploratory or short in duration, with few phase 2 studies. Critical endpoints like villous atrophy, IEL infiltration and anti-TG2 levels are rarely assessed.

## 8. Nutraceuticals

The term nutraceuticals encompasses bioactive compounds from natural or food sources with potential health benefits [190]. Some of these functional compounds include phytochemicals (e.g., polyphenols, terpenoids, alkaloids, etc.), fatty acids, vitamins, minerals or amino acids, among others. Nutraceuticals can exert functions like gliadin sequestration, which prevents its degradation and the subsequent formation of GIPs, a decrease in IP, inhibition of TG2 enzyme or modulation of the gut microbiota, as well as display anti-inflammatory, antioxidant and immunoregulatory effects [191]. Nevertheless, more studies are still needed to elucidate the pathways through which nutraceuticals can exert beneficial effects in the pathophysiology of CeD. A list of studies regarding nutraceuticals and CeD can be found in Table 6 (polyphenol-related strategies) and Table 7 (other bioactives).

### 8.1. Polyphenols

Polyphenols are a type of phytochemical structurally related to phenolic compounds, which possess strong antioxidant properties. Polyphenols can be mainly classified into flavonoids and non-flavonoids. Within the flavonoids, relevant subcategories are flavanols, anthocyanidins, anthocyanins, isoflavones, flavones, flavonols, flavanones and flavanonols. In the non-flavonoid family, relevant sub-types are phenolic acids, stilbenes, lignans and tannins [192]. In the context of CeD, polyphenols can exert beneficial effects through multiple mechanisms. One of them is gliadin sequestration [193], where polyphenols have been hypothesized to be capable of agglomerating and forming 3D structures, which can complex to gliadin and prevent its crossing into the lamina propria and the immune reaction that ensues.

A study on green tea extract, which contains high amounts of polyphenols, confirmed that it was capable of attaching to gliadin in vitro, as seen by an increase in turbidity [194], and, thus, inhibit gliadin digestion. Moreover, in Caco-2 cells, green tea extract’s complexation with gliadin decreased IP and IL-6 and IL-8 secretion. In another study on green tea polyphenols, mainly (-)-epigallocatechin (EGC) and (-)-epigallocatechin-3-gallate (ECGC), it was shown that ECGC, but not EGC, was capable of reducing the in vitro digestion of a gluten + wheat starch mixture, which was attributed to the polyphenol’s crosslinking to both molecules [195].

In a project that aimed to uncover the mechanisms by which polyphenols can have a positive impact in reducing CeD symptomatology, the interaction between the 32-mer GIP and dietary polyphenols (catechin, procyanidin B3, procyanidin C2, EGC and ECGC) was evaluated [196]. The authors reported that procyanidin B3, but, even more so, C2, significantly prevented transport of the 32-mer peptide from the apical to the basolateral side of the intestine using Caco-2 cells. Along the same line, another study evaluated the capacity of polyphenols sourced from artichoke and green tea leaves, as well as cranberries and apples, to complex with gliadin and decrease gliadin-derived immunogenicity and allergenicity [197]. It was found that all but green tea leaves’ polyphenols formed insoluble complexes with gliadin and that cranberry and artichoke extracts did partially prevent basophil degranulation. The same pattern was observed in a study using Raman spectroscopy, which reported conformational changes in gliadin after interaction with the anthocyanins malvin, kuromanin and callistephin [198]; the anthocyanidin cyanidin; the coumarin-derivative 3-ethoxycarbonylcoumarin (3-EcC) [199]; and the flavonoid quercetin [200]. This effect was not observed for the anthocyanins pelargonin, oenin and cyanin [200]. Another study on quercetin [201] reported decreased TG2 and increased glutathione (GSH) and TEER after quercetin treatment alongside pepsin-trypsin-digested gliadin (PT-G) in Caco-2 cells. Quercetin intake in C57BL/6N gliadin-sensitized mice caused a small prevention of the gliadin-associated decrease in the Vh/Cd ratio and a significant decrease in nitric oxide (NO) and TG2 levels but did not affect GSH. Quercetin decreased the % of T-regulatory cells, the T-helper (Th)1/Th2 ratio and many inflammatory cytokines (IL-1β, TNF-α, IL-2 and IL-12p70). An increase in beneficial bacteria such as *B. longum* was also noted.

An alternative study reported that three procyanidins naturally present in grape seeds (i.e., B3, B6 and T2) were all capable of molecularly binding to the 32-mer peptide and that the B6 and T2 compounds strongly prevented 32-mer transport from the intestinal lumen to the lamina propria [202]. Similarly, a project researching the impact of peanut skin proanthocyanidins (PSPc) on CeD showed that treatment of PT–G-sensitized Caco-2 cells with PSPc decreased reactive oxygen species (ROS), TG2, IL-1β, IL-6 and TNF-α levels, especially at high concentrations, indicating a strong antioxidant and anti-inflammatory effect [203]. Moreover, addition of PSPc also increased occludin expression, a marker of intestinal integrity. A very similar experiment was performed using anthocyanin-rich sour cherry extract (AC), where it was reported that AC prevented the gliadin-induced IP. This was concluded after observation that AC intake led to an increase in TEER values, compared to those in the Cacao-2 cells exclusively treated with gliadin, and by a decrease in the permeability coefficient of Lucifer Yellow, a small paracellular marker [204]. Addition of AC alongside gliadin also limited apical TNF-α, IFN-ɣ and IL-8 levels. While ROS levels decreased in the AC + gliadin group, compared to the gliadin-only group, these were still significantly higher than in the control samples.

Propolis is a resin-like substance produced by honeybees, which contains high amounts of polyphenols. The effects of propolis supplementation ex vivo in peripheral blood mononuclear cells (PBMCs) and fecal samples, both from CeD donors, were also evaluated. Culture of PBMCs with ethanolic extract of propolis (EEP) significantly reduced the levels of the oxidative stress markers NO and inducible nitric oxide synthase (iNOS) enzyme and the pro-inflammatory cytokine IFN-ɣ, simultaneously with an increase in the anti-inflammatory cytokine IL-10 [205]. Moreover, it also down-regulated the expression of the nuclear factor kappa-light-chain enhancer of activated B cells (NF-κB) and phosphorylated STAT 3 (pSTAT-3) transcription factors, which can be involved in pro-inflammatory processes. Culture of fecal material from CeD patients with propolis significantly increased the levels of SCFA and of beneficial bacteria such as *Bifidobacterium* [206].

A study on the protective role of curcumin in vitro using the HCT-116 and -29 human intestinal cell lines reported that pre-treatment with curcumin before gliadin exposure prevented an increase in inflammatory and oxidative markers, such as advanced oxidation protein products (AOPPs), Nicotinamide Adenine Dinucleotide Phosphate (NADPH) oxidase (NOX) and myeloperoxidase (MPO), as well as the levels of multiple inflammatory cytokines (IL-1β, IFN-ɣ, TNF-α…) [207]. A similar study showed both in vitro and in vivo that exposure/administration of resveratrol to gliadin-sensitized Caco-2 and C57BL/6N mice caused a decrease in inflammatory malondialdehyde (MDA) and an increase in activity of the anti-inflammatory superoxide dismutase (SOP) enzyme, as well as of GSH levels [208]. Supplementation with resveratrol also led to a significant decrease in TG2, iNOS and cyclooxygenase-2 (COX-2) expression, an increase in occludin activity and a decrease in IP.

### 8.2. Combination of Polyphenols and Other Bioactives

Some studies have also focused on potential synergistic effects between multiple bioactives. The anti-inflammatory and antioxidant effects of the carotenoid lycopene and the polyphenols quercetin and tyrosol were evaluated in an in vitro model of gliadin and IFN-γ-stimulated RAW 264.7 macrophages [209]. All treatments significantly decreased nitrite (NO_2_^−^) and prostaglandin (PGE_2_) production, iNOS and COX-2 expression and NF-κB, interferon regulatory factor -1(IRF-1) and STAT-1α activation.

Regarding this topic, one food, which naturally presents a combination of health-promoting compounds, is cocoa. The beneficial effects of cocoa as a whole [210,211], as well as of cocoa-derived polyphenols [212,213], theobromine [214,215], caffeine [216] and fiber [217], have been studied individually in health and disease. To this end, procyanidin B2-enriched cocoa extract (PCE) was investigated for its potential preventive role in CeD using an in vitro model of gliadin-sensitized Caco-2 cells [218].

**Table 6:** Polyphenol-containing strategies in CeD.

Strategy	Active Principle	Source	Mechanism of Action	Phase *	Ref.
Polyphenols	Polyphenols	Green tea extract	Gliadin sequestration	In vitro	[194]
	(-)-epigallocatechin and (-)-epigallocatechin-3-gallate	Green tea	Gliadin sequestration	In vitro	[195]
	*Catechin*, *Procyanidin B3*, *Procyanidin C2*, *Epigallocatechin* and *Epigallocatechin Gallate*		Gliadin sequestration	In vitro	[196]
	Polyphenols	Artichoke leaves, cranberries, apples, green tea leaves	Gliadin sequestration	In vitro	[197]
	Kuromanin, Callistephin, Oenin, Cyanin, Pelargonin, Malvin (Anthocyanins)		Gliadin sequestration	In vitro	[198]
	Procyanidin B3, B6 and T2 (Tannins)	Grape seed	Gliadin sequestration	In vitro	[202]
	Quercetin (Flavonoid)		Gliadin sequestration	In vitro	[200]
	Cyanidin (Anthocyanidin)	Coumarin	Gliadin sequestration	In vitro	[199]
	Proanthocyanidins	Peanut skin	Gliadin sequestration	In vitro	[203]
	Anthocyanins	Sour cherry extract	Immune and intestinal permeability modulation	In vitro	[204]
	Flavonoids	Propolis dry extract ESIT 12^®^	Immune and gut microbiota modulation	Ex vivo	[205,206]
	Curcumin (Diarylheptanoid)	Turmeric (*Curcuma longa*)	Immune modulation	In vitro	[207]
	Resveratrol (Stilbene)	Grapes, red wine	Immune, intestinal permeability and gut microbial modulation	In vitro and in vivo	[208]
Combination of polyphenols & other bioactives	Lycopene (carotenoid), quercetin (flavonoid) and tyrosol (phenolic alcohol)	Tomatoes/Onions, extra virgin olive oil (EVOO), broccoli/white wine, EVOO	Immune modulation	In vitro	[209]
	Procyanidin B2, theobromine, caffeine (alkaloids)	Cocoa extract	Immune modulation	In vitro	[210]
	Epicatechin (flavanol), theobromine (alkaloid)	Chocolate	Immune modulation	Pilot study, ECCEL2 nº 43.18: 4, 2018	[211]

* ECCEL2 = Ethics Committee of the Comitato Etico Lazio 2.

The results showed that both theobromine and caffeine, individually, significantly decreased TG2 levels (40–60%) and that the reduction was even higher when using the PCE (77% reduction). The addition of PCE to the sensitized model led to a significant decrease in many inflammatory biomarkers, including TG2, COX-2, IL-15, IL-1β, IL-6 and IL-8 levels. Along the same line, another study evaluated the lymphocyte-to-monocyte (LMR) and the platelet-to-lymphocyte (PLR) ratios in CeD patients with habitual dark chocolate consumption. The results showed that they displayed a higher LMR compared to non-chocolate-consuming controls [219]. Since an association between low LMR and autoimmune disease has previously been reported [220], this finding could indicate a beneficial role of chocolate consumption in CeD. Another preclinical study using DQ8-D^d^-villin-IL-15tg mice with predisposition to CeD reported that cocoa supplementation alongside gliadin managed to prevent the increase in anti-gliadin and anti-TG2 Abs, the latter only in male animals, where a decrease in fecal humidity was also found. Cocoa intake also prevented an increase in villi width, induced a change in β-diversity and an increase in beneficial probiotic species such as *Akkermansia muciniphila* or the decrease in the opportunistic genera *Chlamydia*, indicating the potential multispectral effect of cocoa intake in the context of CeD [221,222].

### 8.3. Vitamins

Another group of bioactive compounds is vitamins. These have long been studied in relation to CeD, since Vitamin B deficiency is a common trait of this disease. As far back as 1944, one study reported an improvement in weight gain and height in children with CeD after supplementation with Vitamin B complex [223]. Many studies have researched the vitamin status of CeD patients, mostly reporting deficiencies in folate [224,225], vitamin D and A, and a tendency to lower B_12_ [226], which tends to normalize after gluten exclusion from the diet. Nevertheless, only few works have investigated the potential effect of vitamin supplementation on CeD pathogenesis. One study evaluated Vitamin D3’s (1,25-dihydroxy vitamin D3, VD3) TJs modulation properties in both PT-G-sensitized Caco-2 cells and BALB/c mice [227]. The results of simultaneous application of PT-G and VD3 for 7 days to Caco-2 cells showed an increase in TEER, occludin, claudin-1 and ZO-1 values and a decrease in zonulin expression. In mice, a decrease in serum FITC-dextran 4000 (FD-4) flux after VD3 intake also indicated an improvement in the integrity of the intestinal barrier, which was supported by increases in the relative levels of occludin, claudin-1 and ZO-1. In another study, CeD seronegative patients on a GFD were supplemented with 400 mg of vitamin E, twice daily, for a period of 3 months (KBET/174/B/2013) [228]. The authors reported that, although no significant difference in Vitamin E levels was found before and after supplementation, serum NO and alanine aminotransferase (ALT) levels, as well as glutathione peroxidase-3 (GPx3) activity, did decrease, while Vitamin D levels increased, altogether indicating a decrease in oxidative stress and inflammation.

### 8.4. Fatty Acids

Polyunsaturated fatty acids (PUFAs), especially omega-3 (*n*-3) fatty acids (FAs), have many recognized health benefits, mainly due to their capacity to exert anti-inflammatory effects through the regulation of T cells, macrophages and other immune cells [229]. While multiple studies have evaluated the characteristic FA levels in CeD, only two of them have focused on the effect of FA administration in CeD pathophysiology. A characteristic feature of CeD is villi atrophy, mainly driven by the acquisition of a cytotoxic phenotype by IELs. Since this is mediated by the release of arachidonic acid (AA), which is dependent on cytosolic phospholipase A_2_ (cPLA_2_), one study aimed to assess if the *n*-3 long-chain PUFA docosahexanoic acid (DHA) was able to down-regulate the release of AAc by IECs [230]. Results from the study showed that the addition of DHA to a PT-G-sensitized-Caco2 monolayer effectively decreased the release of multiple inflammatory markers: cPLA2, AAc, COX-2 and PGE_2_. This prevented the establishment of an inflammatory milieu, as observed by a decreased secretion of the pro-inflammatory cytokine IL-8.

In a clinical study (Ethics Committee of the Assis Gurgacz University Center number 2,315,783), CeD patients followed a GFD supplemented with fish oil or the latter and exercise for 12 weeks [231]. The results showed a decrease in C-reactive protein and IL-6 in the group that included exercise.

### 8.5. Terpenes

Terpenes are a type of phytochemical widely found in plants, especially in essential oils but also in some animals and microorganisms. The oil from black cumin seed (*Nigella sativa*, NS) was studied for its potential beneficial effect in the context of RCeD, due to its high content of thymoquinone and monoterpenes [232]. The authors reported that the group with NS intake alongside a GFD in a 450 mg dose daily achieved a significantly higher histological remission than the group that exclusively followed a GFD without supplementation.

### 8.6. Glucosinolates/Isothiocyanates

Glucosinolates are sulfur- and nitrogen-containing compounds present in plants from the Brassicaceae family. At consumption, glucoraphanin, the main glucosinolate found in broccoli, is transformed to sulforaphane, an isothyocyanate-type compound with many reported health benefits but low stability. Hence, a study was performed in a Caco-2 model of inflammation, oxidation and gliadin sensitization to analyze the potential effects of glucoraphanin intake [233]. The results showed that the bioactive compound sulforaphane prevented the secretion of monocyte chemoattractant protein-1 (MCP-1), C_X_C motif chemokine ligand 10 (CXCL10) and IL-8, typically upregulated during inflammation. Moreover, sulforaphane modulated the activity of NF-κB but was not capable of preventing a decrease in TEER values.

### 8.7. Algae

The effect of algae as an adjuvant to the diet is being investigated for multiple diseases, including cardiovascular diseases like obesity or diabetes [234]. One study analyzed the effect of the addition of *Chlorella pyrenoidosa* in a gut simulator fed with the feces of either a healthy subject or a CeD patient following a GFD. The results showed a difference in β-diversity abundance between the CeD and healthy samples [235]. The addition of *C. pyrenoidosa* caused an increase in the CeD sample of the generally recognized as beneficial genera *Faecalibacterium*, *Bifidobacterium* and *Megasphaera* and a decrease in the abundance of the Enterobacteriaceae family, which contains opportunistic bacteria. Moreover, *C. pyrenoidosa* supplementation was linked to an increase in butyrate and propionate. A strong limitation of this study is that only one sample was used for each group (*n* = 1).

### 8.8. Limitations and Strengths of Current Nutraceutical Strategies

Strengths:

- Wide range of bioactive effects: Nutraceuticals offer multiple mechanisms of action relevant to CeD pathogenesis, including gliadin degradation or sequestration, inhibition of TG2 enzyme activity, modulation of IP, anti-inflammatory and antioxidant effects and gut microbiota modulation.
- Safety and tolerability: Since nutraceuticals are naturally occurring, they are generally recognized as safe and are well tolerated, even at higher doses.
- Complementary to existing therapies: Generally, these strategies do not aim to replace a GFD but rather enhance its effectiveness by improving symptom control, supporting mucosal healing and decreasing gluten-derived inflammation.
- Mode of delivery: Most treatments are designed to be delivered orally.

Limitations:

- Predominance of in vitro and preclinical evidence: Most data originated from in vitro and animal models, with only a few human trials.
- Low bioavailability of certain compounds: Some nutraceuticals suffer from poor stability or absorption, limiting their in vivo efficacy unless specially formulated. This remains a challenge for translation into real-world therapies.
- Confounding from GFD adherence: Several studies are conducted in patients already on a GFD, making it difficult to isolate the effect of nutraceutical intervention from the baseline benefits of dietary gluten exclusion.
- Heterogeneity of compounds and protocols: The category “nutraceuticals” includes vastly diverse compounds, delivery forms, and dosages, making standardization, reproducibility, and comparison between studies difficult.

**Table 7:** Non-polyphenol nutraceuticals in CeD.

Strategy	Active Principle	Source	Mechanism of Action	Phase *	Ref.
Vitamins	Vitamin D, 1,25-dihydroxy vitamin D3		Immune and intestinal permeability modulation	In vitro, in vivo, clinical trial, CIEC 53,043,469/050.04–52	[226,227]
Fatty acids	DHA		Immune and intestinal permeability modulation	In vitro	[230]
	DHA and EPA	Fish Oil		Clinical trial, CEP-FAG 2,315,783	[231]
Terpenes	Thymoquinone and monoterpenes	Black cumin (*N. sativa*) oil	Immune modulation	Clinical trial, ECCM-UB	[232]
Glucosinolates/ isothiocyanates	Glucoraphanin/Sulforaphane	Broccoli sprouts (*B. oleracea* var. italica Planck)	Immune and intestinal permeability modulation	In vitro	[233]
Algae	*C. pyrenoidosa*	*Chlorella* sp. (algae)	Gut microbial modulation	Ex vivo	[234]

* CIEC = Clinical investigations ethics committee, ECCM-UB = Ethics Committee of College of Medicine/ University of Baghdad CEP-FAG = Ethics Committee of the Assis Gurgacz University Center.

## 9. Food Modifications

Multiple strategies have aimed to prevent CeD through alterations in gluten-containing foods. To this end, a wide range of approaches have been essayed, from gluten modification to decrease its toxicity to its removal from naturally gluten-containing foodstuffs, like wheat flour, or its processed-derivatives like bread or pasta.

### 9.1. Bacterial and Enzymatic Degradation of Gluten-Contaning Foodstuffs

In order to obtain gluten-free foods, a frequent method used has been the pre-treatment of wheat with either gluten-degrading enzymes or bacteria [236]. Hence, some of the microorganisms and enzymes described in Section 2 and Section 7 have been redirected to food-processing purposes, like AN-PEP, which has been reportedly used for the obtention of gluten-free beers [237] and doughs [238] or papain and alcalase [236], which displayed gluten-hydrolyzing activity while maintaining optimal rheological properties in bread. Two other commercially available proteases, Flavourzyme (*A. oryzae*-containing) and Protamex (*Bacillus lichenformis* and *B. amyloliquefaciens*), were evaluated for their gluten-digesting properties in both wheat flour and vital wheat gluten, demonstrating up to 30% of gluten hydrolysis for Flavourzyme and 15% for Protamex [239]. A subsequent study evaluating the gluten-degrading capacity of three alternative preparations, Flavourzyme 100L, Bioprase SP-20FG (*B. lichenformis*) and Thermoase PC10F (*B. thermoproteolyticus*), validated the prior results, with Flavourzyme again displaying the highest gluten-degrading activity and achieving over 40% hydrolyzation of wheat gluten after 500 min [240]. ASP, DPPIV and EP-B2, alone or together, successfully degraded from 22 to 98% of the gluten present in whole wheat [23]. In another work, CeD patients on a GFD received either regular wheat flour or lactobacilli and fungal protease-pre-digested wheat gluten [241]. Lactobacilli used included *L. alimentarius* 15M, *L. brevis* 14G, *L. sanfranciscensis* 7A and *L. hilgardii*, while fungi were *A. oryzae* and *A. niger*. The results showed that hydrolyzed wheat did not cause an increase in IFN-ɣ levels in CeD patients, contrary to regular wheat. Another study reported that exposure of Caco-2 cells to *L. paracasei* CBA L74-fermented rice flour alongside the 33-mer (P31–43) peptide from α-gliadin significantly reduced its entrance into the basolateral side [242]. Other studies have also used such microorganisms to improve quality, nutritional or rheological properties from gluten-free foods [243,244].

### 9.2. Gluten Genetic Modifications

An alternative strategy that has been investigated is that of gluten modification before its use to generate processed foods. This can be achieved through mutation breeding, γ-irradiation, line deletion, transgenic modifications, gene silencing or targeted mutagenesis [245]. Along this line, one approach to reduce gluten content in wheat and other cereals has been through breeding wheat cultivars with naturally reduced gluten amounts or which display lower immunogenicity [246]. One study reported that deletion of the *Gli-2* loci in wheat, which encodes for 49 α-gliadin expressing genes, removed the presence of 33-mer [247]. Parallelly, another project obtained hypoimmunogenic gluten through gene editing techniques [248]. The authors designed six single-guide RNA sequences to target both α- and γ-gliadins, which contain immunoreactive epitopes, through the CRISPR/Cas9 technology. The results showed increased efficiency and precision as compared to that obtained with γ-irradiation, a method which is accepted in the European Union, contrary to genetic modification [249]. Gene silencing is a post-transcriptional mechanism achieved through RNA interference, where the complementary messenger RNA is degraded, thus preventing the production of targeted proteins [250]. This process was used to down-regulate the expression of gliadins from bread wheat. The results obtained showed a decreased T-cell response from cells obtained from CeD patients’ damaged intestinal tissue [251].

### 9.3. Gluten Transamidation

Since one of the main steps in catalyzing the gluten-derived immune response is deamidation of the glutamine residues present in gliadins, some efforts have focused on preventing this process. Transamidation, contrary to deamidation where the amide group from glutamine is removed, does not lead to the elimination of the amide but its substitution with an amine group. To this end, for this process to occur, both an amine donor, such as a lysine, and a transamidating enzyme, i.e., a microbial or animal transglutaminase, are needed. Several studies have demonstrated that transamidation of gliadin or flour leads to decreased immunogenicity, observed by a smaller IFN-γ response by CeD-biopsy-derived gliadin-specific T-cell lines, as well as a decreased binding affinity to HLA-DQ2 by transamidated gliadins [252]. Similar results have been observed in Caco-2 cells, bone-marrow-derived dendritic cells and BALB/c mice, where transamidated gliadin achieved reduced intestinal damage and permeability, as well as decreased levels of pro-inflammatory cytokines, including IL-6, IL-8 and TNF-α [250]. Moreover, a clinical study with CeD patients in a GFD, whom either consumed regular or transamidated gliadin (Ethical Committee of San G. Moscati Hospital OsSc registry n.06/09, trial n.234, and Ethical Committee ASL Salerno, OsSc registry n.318; trial n.118/AA.GG), reported significant decreases in clinical relapse, intestinal permeability, anti-transglutaminase IgA levels, Vh/Cd and IFN-γ expression in the group with transamidated gliadin intake [253]. As a result, a few studies have investigated the production of bread with decreased immunoreactivity through the use of microbial transglutaminase [254,255].

### 9.4. The Gluten Friendly™ Technology

Multiple studies have been performed using the registered Gluten Friendly (GF)™ technology, which aims to obtain gluten-detoxified flours through structural modifications of gluten proteins, which then do not trigger the regular gluten-derived immune reaction [256]. Briefly, this technology mixes physical and chemical modifications of the wheat kernels through cycles of controlled heat, humidity and resting, which then induces conformational changes that make the gliadins potentially unrecognizable by the immune system [257]. A clinical trial was developed, which consisted of the intake of 3 or 6 g of GF/day (or none in the placebo group) for 12 weeks (NCT03137862) [258]. While the results of the study showed similar Vh/Cd or %IELs infiltration between the three groups, a significant limitation of the study is the lack of a positive control group. While a simultaneous clinical trial, consisting of the administration of none, low (GF bread ~1.5 g), medium (3 g) or high (6 g) daily gluten amounts for 2 weeks (NCT03168490) [259], was completed, results have not been made available.

### 9.5. Limitations and Strengths of Current Gluten-Containing Food Modifications

Strengths:

- Source targeting: These strategies aim to prevent the immune response from initiating by modifying gluten before ingestion, with clear health and economic benefits.
- Potential to improve quality of life: By detoxifying gluten in common foods like bread and pasta, these methods could enhance dietary flexibility, reduce cross-contamination risks, and improve patient adherence and satisfaction.
- Cost-effective and scalable: Compared to pharmaceutical development, food-processing interventions are relatively inexpensive, can be integrated into existing manufacturing systems, and scale efficiently, making them a viable solution in broader populations and low-resource settings.
- Safety and tolerability: Most studies report that modified gluten products are well tolerated in CeD patients, with no major adverse effects or symptom relapses when consumed under controlled conditions. This supports their potential for safe dietary integration, although more safety studies should be performed.
- Mode of delivery: By incorporating modified gluten directly into commonly consumed foods (like bread, pasta, or flour), these strategies offer a non-invasive, patient-friendly alternative to pills or injections. This enhances patient acceptability and compliance, especially for long-term management.

Limitations:

- Predominance of preclinical evidence: Most data originate from in vitro and animal models, with only a few human trials. Many promising findings lack validation in clinical settings, especially with well-defined CeD endpoints like villous atrophy or IEL counts.
- Incomplete or inconsistent detoxification: Not all methods fully remove or neutralize gluten peptides, which may still trigger an immune response in sensitive individuals. Moreover, gluten modification methods may differ in effectiveness depending on the food matrix, processing conditions, and enzyme source, making it challenging to standardize protocols for broad use.
- Mode of delivery: Although modified gluten foods offer a convenient route of delivery, they still require CeD patients to consume specialized or alternative food products. This contrasts with pharmaceutical approaches (like enzymes or immune modulators), which may one day allow patients to eat unmodified gluten-containing foods, potentially offering greater freedom and social normalization.
- Regulatory and consumer acceptance barriers: Strategies involving genetic modification (e.g., CRISPR/Cas9, RNA interference) face regulatory hurdles in many regions (notably the EU), as well as potential consumer resistance.
- Limited clinical validation: Most strategies have not progressed beyond small trials or preclinical models, leaving efficacy in real-world patients uncertain.

## 10. Conclusions

Despite decades of research and the rising global prevalence of CeD, the therapeutic landscape remains remarkably limited. A strict GFD is still the only approved treatment, yet it is both socially restrictive and clinically insufficient for many patients. Numerous alternative strategies, ranging from gluten-degrading enzymes and permeability modulators to immune tolerance therapies and microbiota-targeted interventions, have been explored. However, none have yet demonstrated consistent, reproducible efficacy or proven to be viable, long-term substitutes for a GFD.

What emerges from this study is a sense of insufficiency and urgency. While a lot of work is being conducted and has been carried out on the topic for the last two to three decades, a feasible treatment for CeD still remains out of reach. This brings leads to the question of where the problem lies: Have sufficient efforts been targeted on CeD? Is there a lack of investment? Are we focusing on the wrong pathways? Should we try to avoid the current fragmentation in therapeutic efforts? A pattern that seems to be repeating itself is that, initially, many approaches show promise in early-stage trials or preclinical models but then fail to translate into substantial, lasting benefits in humans. Treatments like enzyme therapies, immune modulators and tolerization vaccines have yielded inconsistent or underperforming outcomes, often hampered by issues of low bioavailability, modest effect sizes or safety concerns.

At the core of this challenge is the complex, multifactorial nature of CeD, which involves not only adaptive and innate immune responses but also gut barrier dysfunction, environmental triggers and the gut microbiome alteration. This complexity makes it unlikely that a single therapeutic strategy will offer a universal solution. In conclusion, although scientific advances have greatly expanded our understanding of CeD pathophysiology and therapeutic targets, we are still far from delivering a practical, scalable treatment. Hence, despite all the efforts focused on this research field, the only current treatment for CD is not any of the abovementioned strategies, but it still remains a lifelong string adherence to a GFD. This underscores the urgent need for better-designed trials, more collaborative efforts, investment and patient-centered outcomes, as well as focus on multimodal therapies that move beyond theoretical benefit and towards real, meaningful improvements in daily life for patients with CeD.

## Figures and Tables

**Table 2:** Gluten neutralization and intestinal permeability modulation strategies.

Strategy	Active Principle	Name	Phase, Clinical Trial	Sponsor	Ref.
Gluten neutralization	Anti-gliadin IgY from the egg yolk of hypersensitized hens	AGY-010	Phase 1 (NCT01765647), Phase 2 (NCT03707730)	Igy Inc.	[53,54,55]
	(P(HEMA-co-SS)) copolymer	BL-7010	Phase 1 and 2 (NCT01990885)	BioLineRx, Ltd.	[56,57,58]
Intestinal permeability modulators	Octapeptide inhibitor of paracellular permeability (TJs modulator)	Larazotide acetate (AT-1001)	Phase 1 (NCT00386165, NCT00386490), Phase 2 (NCT00362856, NCT00492960, NCT00620451, NCT00889473, NCT01396213), Phase 3–terminated (NCT03569007)	9 Meters Biopharma, Inc.	[59,60,61,62,63]

## Abbreviations

The following abbreviations are used in this manuscript:

3-EcC	3-ethoxycarbonylcoumarin
AA	Alopecia areata
AAc	Arachidonic acid
Abs	Antibodies
AFU	Active fluorescent units
AGY	Immunoglobulin Y antibody from the egg yolk
ALT	Alanine aminotransferase
AOPP	Advanced oxidation protein product
APCs	Antigen presenting cells
ASP	*Aspergillus niger*
BF	Bromelain and ficin
CCL	CC motif chemokine ligand
CCR	CC motif chemokine receptor
CeD	Celiac disease
CF	Cystic fibrosis
CFTR	Cystic fibrosis transmembrane conductance regulator
CFU	Colony-forming units
COX-2	Cyclooxygenase-2
cPLA2	Cytosolic phospholipase A2
CXCL	CXC motif chemokine ligand
DGP	Deamidated gluten peptides
DH	Dermatitis herpetiformis
DHA	Docosahexanoic acid
DPPIV	Dipeptidyl peptidase IV
ECGC	(-)-Epigallocatechin-3-gallate
EEP	Ethanolic extract of propolis
EGC	(-)-Epigallocatechin
ELISA	Enzyme-linked immunosorbent assay
EoE	Eosinophilic esophagitis
FA	Fatty acid
FD-4	FITC-dextran 4000
FM PEP	*Flavobacterium meningosepticum*
GCD	Gluten-containing diet
GFD	Gluten-free diet
GIP	Gluten immunogenic peptide
GIT	Gastrointestinal tract
GPx3	Glutathione peroxidase-3
GSH	Glutathione
GSRS	Gastrointestinal symptoms rating scale
HDL	High-density lipoprotein
HLA	Human leukocyte antigen
HPLC	High-performance liquid chromatography
IBS	Inflammatory bowel disease
IBS-D	Diarrhea-predominant inflammatory bowel disease
IEC	Intestinal epithelial cell
IEL	Intestinal epithelial lymphocyte
IFN	Interferon
Ig	Immunoglobulin
IL	Interleukin
iNOS	Inducible nitric oxide synthase
IP	Intestinal permeability
IRF	Interferon regulatory factor
JAK	Janus kinase
LAMA	Lactulose to mannitol
LMR	Lymphocyte-to-monocyte ratio
MasCAM	Mucosal addressin cell adhesion molecule
MCP	Monocyte chemoattractant protein
MDA	Malondialdehyde
MHC	Major histocompatibility complex
MoAb	Monoclonal antibody
MPO	Myeloperoxidase
MX PEP	Myxococcus xanthus
*n*-3	Omega-3
NADPH	Nicotinamide Adenine Dinucleotide Phosphate
NCGS	Non-celiac gluten sensitivity
NF-κB	Nuclear factor kappa-light-chain enhancer of activated B cells
NK	Natural killer
NKT	Natural killer T cells
NO	Nitric oxide
NOD/DQ8	HLA-DQ8 non-obese diabetic
NOX	NADPH oxidase
P(HEMA-co-SS)	Poly(hydroxyethyl methacrylate-co-styrene sulfonate)
PBMC	Peripheral blood mononuclear cells
PCE	Procyanidin B2-enriched cocoa extract
PGE2	Prostaglandin E2
PLR	Platelet-to-lymphocyte ratio
PSPc	Proanthocyanidins
pSTAT	Phosphorylated STAT
PT-G	Pepsin-trypsin-digested gliadin
PUFA	Polyunsaturated fatty acid
RCeD-II	Type II refractory CeD
ROS	Reactive oxygen species
SCFA	Short-chain fatty acids
SIRT6	Sirtuin 6
SOP	Superoxide dismutase
Srp	Serine protease inhibitor
STAT	Signal transducer and activator of transcription
TEAE	Treatment-emergent adverse event
TEC	Tyrosine kinase expressed in hepatocellular carcinoma
TEER	Transepithelial electrical resistance
tg	Transgenic
Th	T-helper
TLR	Toll-like receptor
tTG2	Tissue transglutaminase 2
VD3	Vitamin D3
Vh/Cd	Villous height/crypt depth ratio
